# DNA methylation regulator-mediated modification patterns and risk of intracranial aneurysm: a multi-omics and epigenome-wide association study integrating machine learning, Mendelian randomization, eQTL and mQTL data

**DOI:** 10.1186/s12967-023-04512-w

**Published:** 2023-09-23

**Authors:** Aierpati Maimaiti, Mirzat Turhon, Aimitaji Abulaiti, Yilidanna Dilixiati, Fujunhui Zhang, Aximujiang Axieer, Kaheerman Kadeer, Yisen Zhang, Aisha Maimaitili, Xinjian Yang

**Affiliations:** 1 Department of Neurosurgery, Xinjiang Medical University Affiliated First Hospital, Urumqi, Xinjiang 830017 People’s Republic of China; 2 Department of Interventional Neuroradiology, Beijing Neurosurgical Institute, Capital Medical University, 100070 Beijing, People’s Republic of China; 3 Department of Interventional Neuroradiology, Beijing TianTan Hospital, Capital Medical University, Beijing, People’s Republic of China; 4 Xinjiang Medical University, Urumqi, Xinjiang People’s Republic of China

**Keywords:** Intracranial aneurysms, DNA methylation regulator, Multi-omics, Machine learning, Genome-wide association studies, Mendelian randomization

## Abstract

**Background:**

Intracranial aneurysms (IAs) pose a significant and intricate challenge. Elucidating the interplay between DNA methylation and IA pathogenesis is paramount to identify potential biomarkers and therapeutic interventions.

**Methods:**

We employed a comprehensive bioinformatics investigation of DNA methylation in IA, utilizing a transcriptomics-based methodology that encompassed 100 machine learning algorithms, genome-wide association studies (GWAS), Mendelian randomization (MR), and summary-data-based Mendelian randomization (SMR). Our sophisticated analytical strategy allowed for a systematic assessment of differentially methylated genes and their implications on the onset, progression, and rupture of IA.

**Results:**

We identified DNA methylation-related genes (MRGs) and associated molecular pathways, and the MR and SMR analyses provided evidence for potential causal links between the observed DNA methylation events and IA predisposition.

**Conclusion:**

These insights not only augment our understanding of the molecular underpinnings of IA but also underscore potential novel biomarkers and therapeutic avenues. Although our study faces inherent limitations and hurdles, it represents a groundbreaking initiative in deciphering the intricate relationship between genetic, epigenetic, and environmental factors implicated in IA pathogenesis.

**Graphical Abstract:**

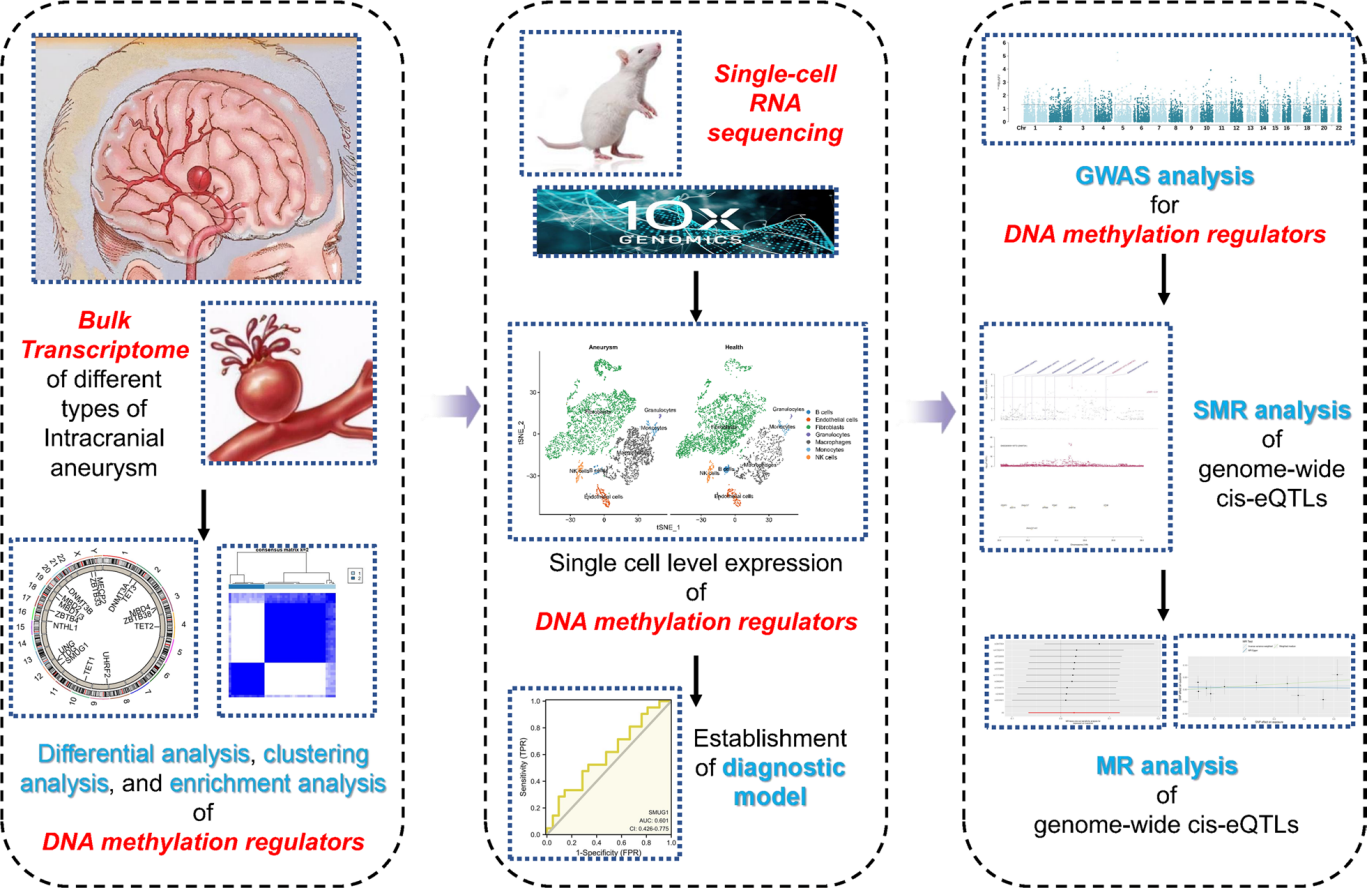

**Supplementary Information:**

The online version contains supplementary material available at 10.1186/s12967-023-04512-w.

## Introduction

Intracranial aneurysms (IAs), which typically form at the bifurcation of intracranial arteries, are relatively prevalent and life-threatening conditions. This disorder includes both ruptured and unruptured aneurysms, affecting ~ 3.2% of the population [1]. When an IA ruptures, it can cause subarachnoid hemorrhage (SAH) [2], a particularly severe stroke subtype that accounts for 5–10% of all stroke cases in the United States [3]. Aneurysmal SAH has a high mortality rate, and survivors often experience chronic neurophysiological events and a diminished quality of life [4, 5]. At present, there is no optimal treatment strategy for IA. Neurosurgeons must evaluate numerous aneurysm-specific and patient-specific risk factors, including aneurysm morphology, hemodynamics, patient age, symptoms, and comorbidities, as well as the potential risks associated with treatment. Currently, the repertoire of therapeutic strategies for intracranial aneurysms encompasses surgical clipping and a diverse array of endovascular approaches. These include endovascular coiling, bypass procedures, and flow-diverts. Collectively, these techniques represent the contemporary landscape of IA treatment modalities within the realm of cerebrovascular medicine [5].

However, the optimal treatment modality and timing for intervention for both ruptured and unruptured intracranial aneurysms (UIAs) remain a topic of debate [6]. Additionally, the primary intervention method involves invasive surgery, which carries the risk of various complications. Consequently, effectively managing UIAs and treating IAs remain a significant clinical challenge. Therefore, it is essential to enhance our understanding of IA pathogenesis to develop viable treatment strategies for this condition.

Notable progress has been achieved in the realm of epigenetics concerning IA. Epigenetics encompasses processes such as DNA methylation and reversible protein modifications (e.g., histones), including acetylation, which independently modulate gene expression beyond the DNA sequence [7]. DNA methylation is a widely studied epigenetic modification that involves the addition of a methyl group to the fifth carbon position of the cytosine DNA nucleotide, leading to the formation of 5-methylcytosine [8, 9]. Governed by both genetic and environmental determinants, DNA methylation serves as a mediator of gene-environment interplay, thereby influencing the susceptibility to a multitude of complex pathologies. This pivotal function is primarily attributed to its integral role in modulating gene expression regulation [10, 11]. Epigenomic association studies (EWAS) have elucidated the correlation between DNA methylation and various phenotypes [12–14].

As a consequence, DNA methylation may function as both a disease biomarker and a contributory factor in its pathogenesis. The identification of genetic loci associated with the methylation of cytosine-phosphate-guanine sites (CpGs), particularly DNA methylation quantitative trait loci (mQTLs), contributes to our understanding of the molecular mechanisms involved in disease associations. Furthermore, this knowledge fosters the establishment of causal inferences concerning the participation of DNA methylation in the pathogenesis of various diseases. Additionally, genome-wide association studies (GWAS) have successfully pinpointed numerous genetic variants linked to disease [15]. Nonetheless, the molecular mechanisms connecting these variants with diseases remain largely elusive. Investigating the co-localization of GWAS-associated genetic variants and mQTL variants could provide insights into the molecular underpinnings of the relationship between genetic variants and diseases [16]. Our hypothesis posits that an analysis of the intersection between mQTL variants and established GWAS disease-associated genetic variants would offer a deeper insight into the collective role of genetic and environmental factors in determining disease susceptibility. Additionally, we aim to expand our comprehension of the biological significance of disease-related CpG sites by co-localizing mQTLs with genetic variants correlated with gene expression, also known as eQTLs [16]. Employing the effect sizes of GWAS on mQTL variants for different diseases, we can conduct causal inference tests to explore the putative causal impacts of CpG on various diseases [17–19].

DNA methylation facilitates heterochromatin assembly and gene suppression [20], while histone acetylation promotes chromatin relaxation, leading to gene transcription [21]. Histone methylation has a broader range of functions, including transcriptional activation (K79, K36, and H3K4) and repression (H4K20, K27, and H3K9) [22]. Lysine has three distinct methylation states (monomethylated, dimethylated, and trimethylated), which are generated by specific enzymes that add [lysine methyltransferases (KMTs)] or remove [lysine demethylases (KDMs)] methyl groups to specific lysine residues of histones [23]. Recent studies have shown that IA patients exhibit lower levels of MAP3K10 methylation, which could potentially serve as a predictor of IA risk, particularly among women [24]. Additionally, DNA methylation and mRNA expression of glutathione S-transferase alpha 4 have been shown to be correlated with IAs in a gender-specific manner [25]. In addition, the DNA methylation of the patatin-like phospholipase domain-containing protein 6 gene has been implicated in contributing to the risk of intracranial aneurysms, especially in males [26]. Moreover, family-based studies have expanded our understanding of the genetic factors involved in familial intracranial aneurysms, with 17 independently verified loci identified across the genome being associated with an increased risk of IA [27]. These findings have emerged mainly from genome-wide association studies, particularly those involving extensive collaborative efforts [27]. Genetic research on IA has been complemented with the MR technique, which has revealed a link between increased genetic predictions of sex hormone binding globulin (SHBG), bioavailable testosterone (BioT) [28], serum calcium (S-Ca), and 25-hydroxyvitamin D (S-25OHD)[29] levels and a higher vulnerability to aneurysmal subarachnoid hemorrhage (aSAH). Additionally, demographic and lifestyle factors such as gender[30], smoking, high-fat diets, hypertension [31] and tobacco and alcohol consumption [32] also appear to influence related traits and increase the risk of IA. Notably, increasing serum magnesium levels has been found to decrease the risk of IA and associated bleeding [33]. Although epigenetic regulatory mechanisms are not yet fully established in the context of IA, epigenetic studies of the disease may contribute to a better understanding of its biology and pathology, as well as identifying potential therapeutic targets.

IA are a complex disease with poorly understood causes, and the role of epigenetic modifications, specifically DNA methylation, in the formation and rupture of IA remains an interesting topic. To address this issue, we conducted a comprehensive analysis of DNA methylation modifiers in IA, with a focus on distinguishing molecular signatures between normal tissue, UIA and ruptured intracranial aneurysms (RIA) samples. Although we did not find any significant correlations between infiltrating immune cell abundance, immune response gene sets, IA, and DNA methylation regulators, we identified distinct m6A modification patterns based on 19 DNA methylation modulators, which were further evaluated in different isoforms. Using machine learning methods, we developed a predictive model for IA rupture based on 17 DNA methylation-related genes (MRGs) in the global IA cohort. Finally, we applied GWAS, SMR, and MR methodologies to demonstrate the critical role of DNA methylation-related genes in the pathogenesis of intracranial aneurysms.

## Materials and methods

### Data pre-processing

Initial filtering of relevant databases using the keywords "IA" and previous literature [1] led us to identify six candidate datasets, namely GSE122897, GSE54083, GSE75436, GSE13353, GSE36791, and GSE159610. From these datasets, a total of 21 unruptured intracranial aneurysm (UIA) samples, 21 ruptured intracranial aneurysm (RIA) samples, and 16 normal intracranial artery (Normal) samples from GSE122897 were selected as the modeling dataset. In addition, 5 UIA samples, 8 RIA samples, and 10 Normal samples for GSE54083, 8 UIA samples and 11 RIA samples for GSE13353, 15 Normal samples and 15 UIA samples for GSE75436, 23 Normal samples and 24 UIA samples for GSE159610, and 43 RIA samples and 18 Normal samples for GSE36791 were included. To eliminate batch effects, transcriptomic data from the three training datasets, namely GSE54083, GSE75436, and GSE13353, were combined using the "combats" approach in the "sva" package, and principal component analysis (PCA) was used to verify the successful removal of the batch effect. A total of 19 methylation-related genes (MRGs) were annotated based on previous literature, including 3 erasers (TET1, TET2, TET3), 3 writers (DNMT1, DNMT3A, DNMT3B), and 13 readers (UHRF2, MECP2, UNG, TDG, NTHL1, SMUG1,MBD1, MBD2, MBD3, MBD4, ZBTB33, ZBTB38, ZBTB4) [34, 35]. The expression of these MRGs between the samples was compared using either the Kruskal–Wallis test or Wilcox test, visualized using the RCircos package to study their chromosomal location, and their protein levels were explored using the STRING database to understand their relationships.

In the present study, we aimed to expand our dataset by incorporating scRNA-seq data from a male mouse brain aneurysm model, GSE193533, which consisted of pre-induction samples (GSM5813881) and aneurysm formation samples (GSM5813883). To ensure the quality of our dataset, we applied strict filtering criteria, such as a threshold of > 3 cells expressing a given gene, > 200 different genes expressed per cell, and < 10% mitochondrial gene expression. We then utilized the Seurat package for cell clustering, with a resolution of 0.5, as previously described. Using the singleR package and CellMarker database, we identified various cell types based on the expression of specific marker genes, including Fibroblasts, Macrophages, NK cells, Endothelial cells, B cells, Granulocytes, and Monocytes. We calculated MRGs scores for each cell type using the mean method and subsequently performed a Kruskal–Wallis test to compare differences between groups.

### Unsupervised clustering analysis

The present study focused on identifying distinct modification patterns based on the expression of MRGs through unsupervised clustering analysis. The number and stability of the resulting clusters were assessed through the employment of the consensus clustering algorithm. K-means clustering was applied iteratively over 100 rounds, with 80% of the samples utilized for each round, to ensure the stability of the clusters. The optimal number of clusters was selected by evaluating the clustering score of the Cumulative Distribution Function (CDF) curve. In addition to this, the reliability of the consensus clustering approach was validated using Principal Component Analysis (PCA).

### Immuno-infiltration analysis

The application of the CIBERSORT tool facilitated the estimation of the abundance of specific infiltrating immune cells. This analytical approach revealed the intricacies of the distribution levels of LM22 immune cells based on gene sets. In order to determine distinctions in the enrichment fraction of immune cells among various modification patterns, we utilized the Wilcox test.

### Pathway enrichment analysis

The gene expression matrix was subjected to a transformation into a score matrix using the Gene Set Variation Analysis (GSVA) algorithm. Following this, the scores attributed to biological signaling pathways were scrutinized for differences based on various methylation modification patterns utilizing the well-established Limma package. A significance threshold of P < 0.05 was applied to ascertain differences.

### Diagnostic marker-based prediction model development and validation

As in the previous literature pipline [36],to develop a proficient classification prediction model for the systematic use of blood to tissues using selected biomarkers, a series of 12 commonly used machine learning algorithms were employed including Lasso, Ridge, Enet, Stepglm, SVM, glmBoost, LDA, plsRglm, RandomForest, GBM, XGBoost, and NaiveBayes. A combination methodology was implemented in the final calculation of the model based on the integration of UIA and RIA sample data sets. To train the model, GSE122897 was selected as the reference dataset while ensuring the elimination of batch effects for tissue samples from GSE54083, GSE75436, GSE13353, and validation data for blood samples from GSE159610 and GSE36791. Thorough composition validation techniques were employed to validate the model performance. The present study developed a classification prediction model for the systematic use of blood to tissues utilizing a combination of 12 commonly used machine learning algorithms. The performance evaluation of the models was conducted by calculating the area under the ROC curve (AUC) for each model and model gene followed by visual representation of the results using heat maps. The optimal model's performance was assessed using calibration curve and decision curve analysis (DCA) techniques.

### Annotation of immune-related characteristics for the MRGs signature

In this study, the authors collected seven types of immune modulators [37] and identified seven immune subtypes and immunophenotypes (IPS) [37, 38].The abundance of infiltrates of different immune cells between UIA and RIA groups was explored through the use of four different algorithms, namely EPIC algorithm, Microenvironmental Cell Population Counter (MCP Counter) algorithm [39], CIBERSORTabs, and the ESTIMATE algorithm [40]. The degree of differences in immune cell subset responses between the two groups under different algorithms was revealed through the use of heat maps and boxplots. These findings provide valuable insights into the immunological differences between the UIA and RIA groups, and may inform the development of more targeted and effective therapeutic strategies for these patients.

### Establishment of a nomogram

Incorporation of 17 MRGs genes and pertinent clinical information (age, sex) aided in the establishment of a nomogram with the aid of the RMS package. The calibration curve was utilized as an assessment tool to determine the accuracy of the nomogram. Further evaluation of the clinical usefulness of the nomogram was conducted through decision curve analysis.

### Constructing potential TF and miRNA target gene regulatory networks

The utilization of the MiRNet online database exposed potential miRNA-targeted diagnostic genes and upstream transcription factors (TFs) for prognostication. The outcome was then rendered perceptible through the use of Cytoscape software.

### GWAS enrichment

Genome-wide association study (GWAS) data was harnessed in this study to detect variants located outside the major histocompatibility complex (MHC) region (GRCh37: 6:28,477,797–33,448,354) and their respective mapping to 19,019 protein coding genes to determine their statistical significance. The Bonferroni correction was adopted to account for multiple testing, and genes with a P value of < 0.05 were considered statistically significant. Moreover, LD information was obtained from the 1 KG dataset for all MAGMA analyses. To identify significantly associated gene sets, MAGMA gene-set analysis was conducted on 15,496 gene sets sourced from the MSigDB v.7.0 database. Gene sets were regarded as significant if the P value was below 0.05, following Bonferroni correction for multiple testing. To facilitate the forward selection strategy of finding associated gene sets, we leveraged cutting-edge genomic techniques such as the MAGMA v.1.08 conditional analysis. Notably, the most statistically significant gene set was chosen as a covariate, while the remaining gene sets were analyzed. Subsequently, this selected gene set was incorporated as an additional covariate in the subsequent round addition, alongside the DNA methylation regulators gene set, and the analysis rerun. This iterative process continued until there was no gene set below the statistical threshold of significance (P < 0.05).

### SMR data source

To select eQTL instruments associated with DNA methylation-related genes, we extracted genetic variants (cis) located within 1000 kb on either side of the coding sequence (cis), which are closely related to gene expression, using eQTL summary statistics obtained from the eQTLGen consortium (https://www.eqtlgen.org/cis-eqtls.html). The eQTLGen consortium provided data on 31,684 trait-associated single nucleotide polymorphisms (SNPs) derived from 10,317 individuals. However, it is worth noting that eQTLGen does not encompass variants associated with gene expression levels located on the X and Y chromosomes. MR cis-mQTL instruments for genetic variants that are closely linked to the selected genes were extracted using pooled data from two cohorts (n = 1980) of meta-analytic studies.

The quality control conditions for instrumental variables are: (1) All SNPs included in the initial analysis had at least suggestive P < 5 × 10^–8^; (2) We removed SNPs with R2 > 0.9 and R2 < 0.05 around the top SNPs and kept only those with R2 ≤ 0.9 among the remaining paired SNPs. (3) Data from the European population of the 1000 Gene project were selected for LD removal from GWAS data (data source: https://ctg.cncr.nl/software/magma). After performing quality control, 133 SNPs associated with the expression of 19 DNA methylation-related transcripts were selected from the cis-eQTL for study. All SNPs included in the initial analysis were at least suggestive at P < 5 × 10^–8^. HEIDI test was the co-localization method for this step using external reference estimation of LD when P-SMR < 0.05 and P-HEIDI > 0.01, we considered the results to be reliable.

GWAS summary statistics for IA, uIA, SAH endings were obtained from publicly available databases (https://figshare.com/articles/dataset/Intracranial_aneurysm_genome-wide_association_study_summary_statistics_2020/11303372). Details of all QTL and GWAS datasets for this study are provided in the Additional file 6 [41] (Download eQTL at: https://www.eqtlgen.org/cis-eqtls.html and mQTL download address: https://yanglab.westlake.edu.cn/data/SMR/LBC_BSGS_meta_lite.tar.gz).

### Principle of SMR

The SMR methodology was developed to explore the pleiotropic associations between genetic traits, such as gene expression, DNA methylation, or protein abundance, and important complex traits like disease phenotype. To ensure adherence to the Mendelian randomization (MR) principles in our analysis, causality was assessed in the following manner: βeQTL − IA/SAH/uIA = βSNP − IA/SAH/uIA/βSNP − eQTL. βeQTL − IA/SAH/uIA was computed as the effect size of the selected genetic variation on genes or traits determined by those genes and the estimated magnitude of effect on IA/SAH/uIA. The βSNP-eQTL represents the estimated influence of SNP on the selected genes or traits determined by those genes (i.e., genetic variation-exposed trait association), and βSNP-IA/SAH/uIA is the estimated effect size of SNP on IA/SAH/uIA (i.e., the same genetic variation-outcome trait association). SMR software for Linux version 1.3.1 was used to execute the SMR with the default option (https://yanglab.westlake.edu.cn/software/smr/#Overview). The OR eQTL-IA/SAH/UIA, which represents the ratio of the selected genes or traits determined by them and IA/SAH/uIA risk, was estimated as follows: OR eQTL-IA/SAH/uIA = exp (βeQTL-IA/SAH/UIA), where OR is the ratio estimate for each 1-ln increase in mitochondrial genome level and exp is the base of the natural logarithm.

### MR

Upon completing the primary SMR analysis, this study conducted a sensitivity analysis using the TwoSampleMR R software package, which featured an additional five MR methods, including MR Egger, weighted median, inverse variance weighted (IVW), simplex, and weighted model. To bolster the robustness of our final outcomes, separate MR analyses were conducted on all SNPs of selected genes and individual genes. The R software (version 4.1.2) was employed for all analyses in this section.

### Statistical analysis

E employed the odds ratio (OR) with 95% confidence intervals (CIs) to estimate effect sizes. Our primary MR analysis utilized the 2-sample IVW method, which was followed by alternative MR methods, namely MR-Egger, weighted median, weighted mode, and simple mode, all of which provide more robust estimates to address potential directional pleiotropy [42]. To obtain IVW estimates, we conducted a meta-analysis of SNP-specific Wald ratios between the exposure and outcome effects, using a random-effects inverse variance method weighted by the standard errors, while accounting for heterogeneity [43]. The present study employed a variety of MR methods, including MR-Egger and weighted median, to address potential issues with directional pleiotropy, where SNPs influence the outcome through pathways that do not involve the modification of the exposure. Weighted median, for instance, facilitates grouping SNPs into clusters and deriving an estimate based on the cluster with the most SNPs, under the assumption that at least half of the SNPs are valid [44]. Heterogeneity tests and sensitivity analyses were conducted to ensure the robustness of the results. The Cochran Q test, followed by MR-Egger and MR-Pleiotropy Residual Sum and Outlier, were used to assess horizontal pleiotropy [45, 46]. The MR was performed again applying a "leave-one-out" sensitivity analysis to identify potentially influential SNPs. In addition, the Steiger-MR approach was employed to test whether the SNPs were responsible for more variance in exposure than outcome, which could indicate reverse causation [47]. R2 values calculated as the sum of 2 * EAF * (1 − EAF) * β2. All analyses were carried out in the R programming language using the "TwoSampleMR" package, and statistical significance was set at P < 0.05.

## Results

### Tissue-level normal, RIA, and UIA samples have differential expression of MRGs

**Fig. 1:**
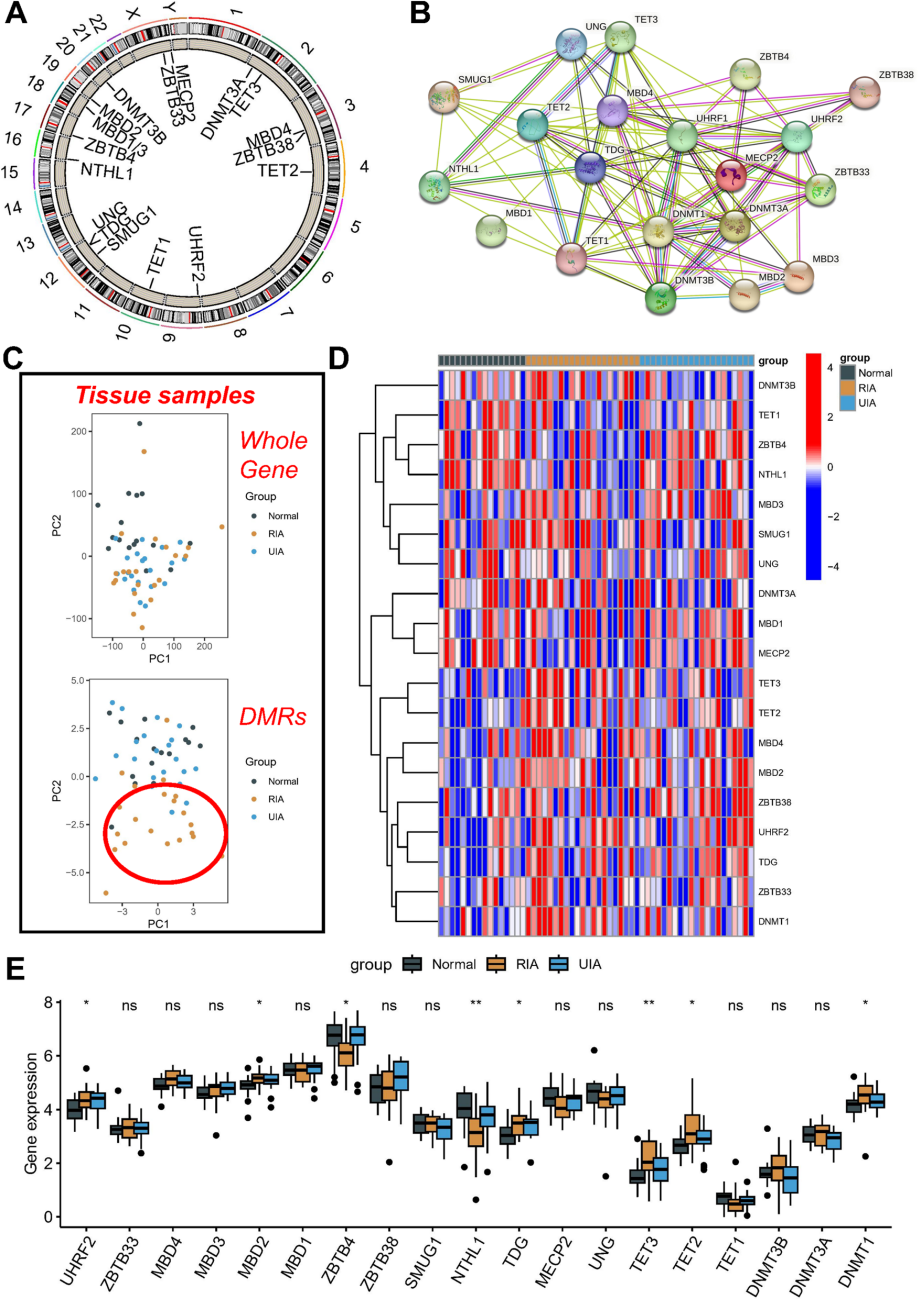
Genetic variations and expression of MRGs in IA tissue samples. **A** Localization of 19 MRGs on the 23 chromosome. **B** PPI analysis of 19 MRGs. **C** At the tissue level, principal component analysis separates normal (grey), RIA (yellow) and UIA samples (blue). Heat map (**D**) and box plot (**E**) showing 19 MRGs differentially expressed in normal, RIA and UIA tissues. *p < 0.05; **p < 0.01; ***p < 0.001; *ns* no statistical significance

The present study aimed to characterize the chromosomal positions of 19 annotatable MRGs, as shown in Fig. 1A. Of these MRGs, UNG, TDG, and SMUG1 were located on chromosome 12, MECP2 and ZBTB33 on chromosome X, and MBD4 and ZBTB38 on chromosome 3, while others were found to be independently located on other chromosomes. To investigate the protein interaction levels of these MRGs, we utilized the STRING database (Fig. 1B), and the analyses showed that they often function as a protein complex (Fig. 1C). The PCA scatter plot representing the whole gene expression profile in the GSE122897 dataset did not reveal any obvious discrete pattern for different samples. However, when PCA typing was performed for the whole differentially methylated regions (DMRs) level, the RIA samples were identified as being independent from the normal and UIA samples, suggesting that the RIA samples may have more specific methylation levels. Accordingly, it can be inferred that the degree of methylation likely plays a key role in the occurrence of IA rupture. The heat map analysis also revealed significant differences in the expression levels of the 19 MRGs among the three different samples. Furthermore, most of the MRGs were significantly more highly expressed in the RIA samples (Fig. 1D). Finally, box plots were generated for a total of eight MRGs exhibiting significant expression differences in these samples, of which MBD2, TDG, TET3, TET2, and DNMT1 were significantly more highly expressed in the RIA samples (Fig. 1E).

In conclusion, MRGs appear to have played a critical role in the development of IA rupture at an organizational level.

### Blood level normal, RIA, UIA samples have differential expression of MRGs

**Fig. 2:**
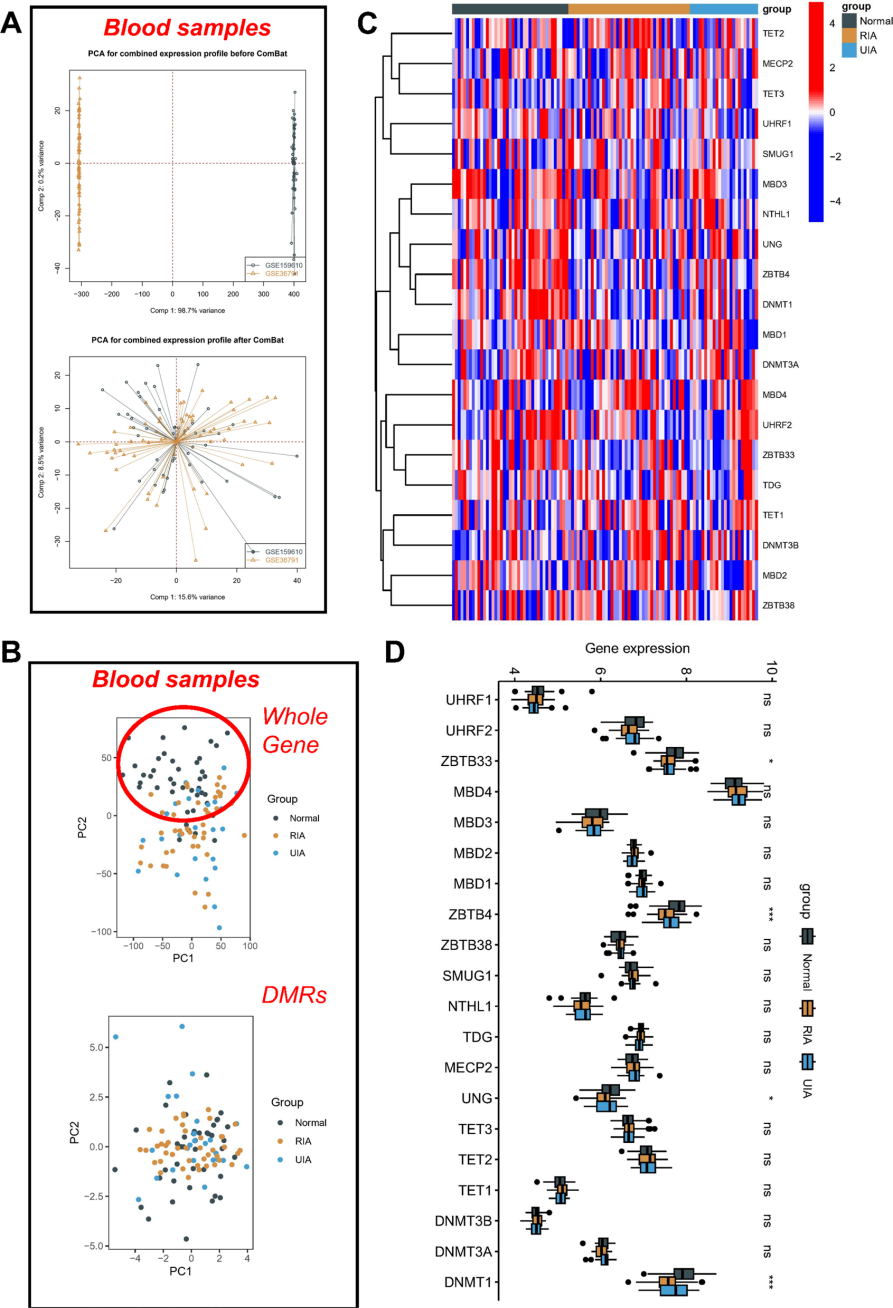
Genetic variations and expression of MRGs in IA blood samples. **A** PCA for combined expression profile before and after ComBat. **B** At the blood level, principal component analysis separates normal (grey), RIA (yellow) and UIA samples (blue). Heat map (**C**) and box plot (**D**) showing 19 MRGs differentially expressed in normal, RIA and UIA blood. *p < 0.05; **p < 0.01; ***p < 0.001; *ns* no statistical significance

The ComBat method was employed to address batch effects in the GSE159610 and GSE36791 datasets of blood samples. Prior to the batch removal, the samples from the dataset were clustered based on the first two principal components (PC) of the non-normalized expression values, as compared to the scatter plot derived from the normalized expression level PCA that exhibited a significant reduction of batch effects resulting from distinct datasets (Fig. 2A). Interestingly, scatter plots of the whole gene expression profile via PCA demonstrated distinct patterns in normal samples compared to RIA and UIA samples, which were intermixed. Remarkably, PCA profiling of the expression profiles of DMRs revealed only a small proportion of discrete UIA samples, whereas the rest remained intermixed (Fig. 2B). Furthermore, the heatmap assay uncovered a minor variation in the expression of the 19 MRGs across the three samples (Fig. 2C), while box line plots specifically detailing only three MRGs, namely ZBTB33, UNG, DNMT1, exhibited significant expression differences across the three different samples (Fig. 2D).

In brief, in the context of peripheral blood, individuals with IA rupture exhibit relatively minor changes in MRGs compared to healthy controls, but manifest greater dysregulation in their whole gene expression profile in comparison to both normal subjects and those with unruptured ICAs.

### Methylation modification patterns mediated by specific MRGs in IA patients

**Fig. 3:**
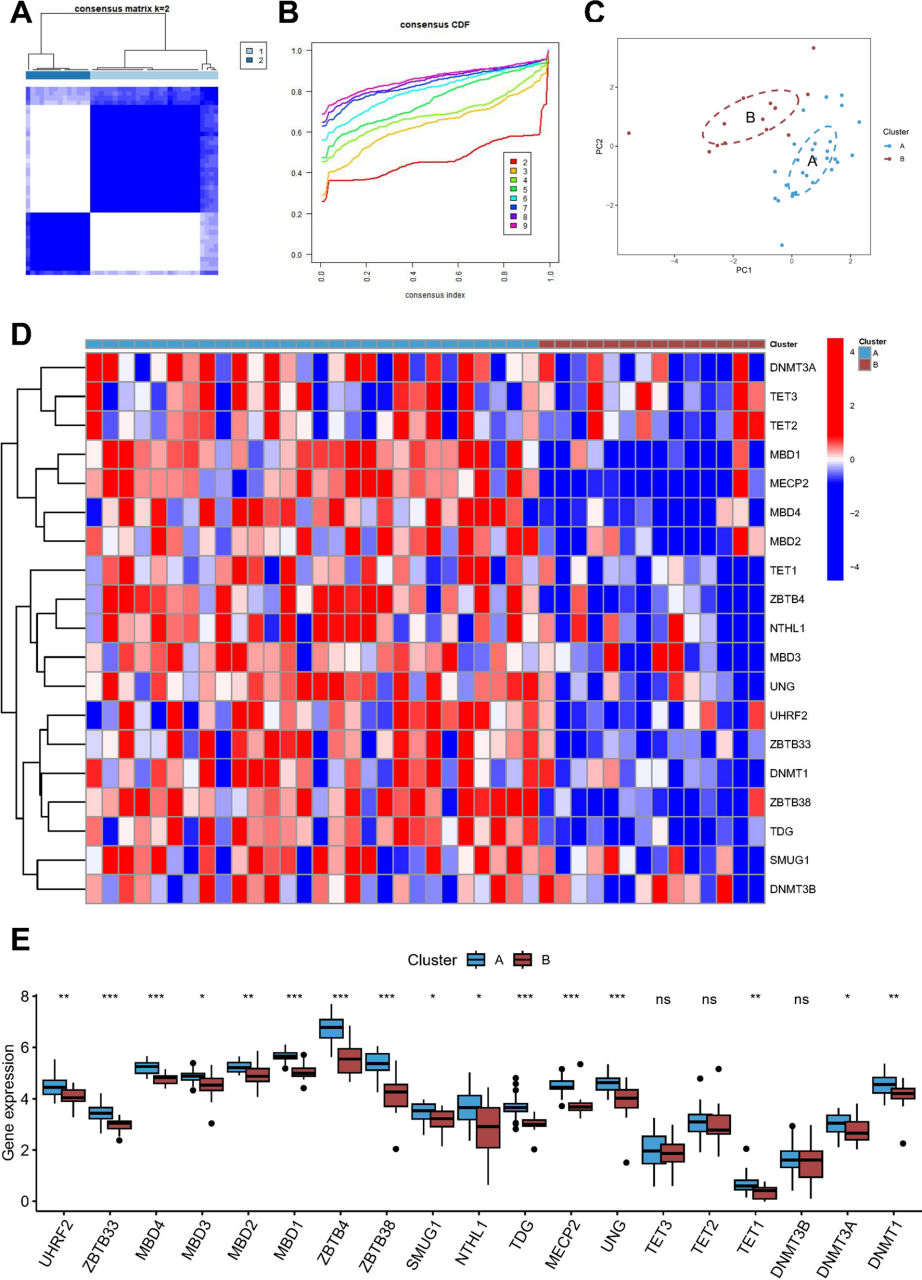
Consensus clustering results in IA cohorts. **A** Consensus clustering matrix of k = 2 as the optimal cluster number. **B** CDF curves of the consensus score from k = 2–9. **C** PCA principal component analysis of two clusters. Each subgroup was distinguished by different colors. Heat map (**D**) and box plot (**E**) showing 19 MRGs differentially expressed in A and B cluster. *p < 0.05; **p < 0.01; ***p < 0.001; *ns* no statistical significance

Given the potential role of MRGs in triggering IA rupture at the tissue level, we carried out a cluster analysis using the 19 MRGs to investigate potential associations between methylation modification patterns and IA in a unsupervised manner. Our k-means analysis (Fig. 3A) area under the curve analyses revealed the identification (Fig. 3B) of two distinct methylation modification isoforms (Fig. 3C). Significantly, the majority of MRGs in subtype A samples showed upregulation, with the exception of TET3, TET2, and DNMT3B (Fig. 3D–E). Notably, among the subtype A samples, 20 out of 28 were RIA samples, whereas only one sample was classified as subtype B (Additional file 1: Fig. S1A). These findings suggest that elevated expression of MRGs could represent a potential methylation modification pattern in IA patients specifically associated with IA rupture and could serve as an important predictive marker of such rupture.

The contribution of immune factors to IA rupture is widely recognized, thus prompting our team to investigate potential differences in IA methylation modifications using the CIBERSORT algorithm. Ineligible samples (P > 0.05) were excluded to ensure the validity of our analysis, and the resulting histogram displays the proportion of various immune cells across different methylation modification patterns (Additional file 1: Fig. S1B). Notably, differential analysis revealed significant differences in Plasma cells between methylation modification patterns, while other cells displayed no significant differences (Additional file 1: Fig. S1C). To further elucidate the impact of the proposed MRGs score on the immune microenvironment, we employed seven immune deconvolution algorithms, namely IPS, EPIC, MCPcounter, CIBERSORT, CIBERSORTabs, xCELL, and ESTIMATE. This step allowed us to ascertain the robustness and reproducibility of our findings, while generating immune landscape profiles for groups A and B (Additional file 2: Fig. S2A). The present study investigated the potential immune cell subsets and corresponding functional pathways involved in A and B groups for APC co-stimulation. Our comprehensive comparative analysis revealed distinct immune cell subsets, including B cells, iDCs, mast cells, neutrophils, NK cells, T helper cells, Th1, Th2, and Treg (Additional file 2: Fig. S2B). Interestingly, our findings suggested that the MRGs scores did not significantly modulate the expression of the majority of immunological checkpoints between the A and B groups (Additional file 2: Fig. S2C), Furthermore, we observed that the different methylation activation status at the IA tissue level may play a crucial role in IA rupture, independently of immune factors (Additional file 2: Fig. S2D). The implications of these findings warrant further investigation regarding the underlying mechanisms of IA pathogenesis.

### Biological properties of different methylation modification patterns

**Fig. 4:**
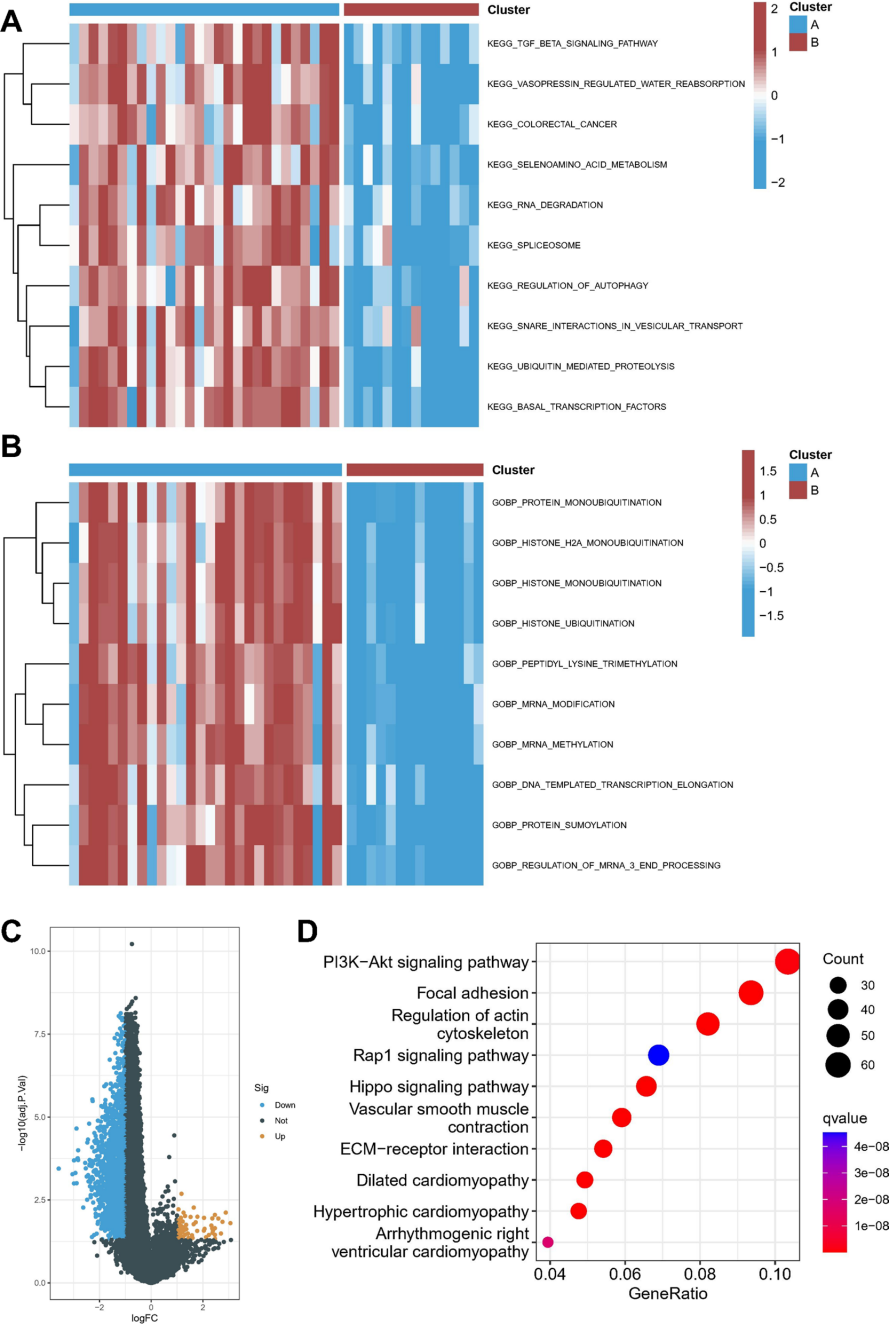
Biological function in two clusters. **A** Heatmap of matrix of KEGG enrichment scores using GSVA algorithm. **B** Heatmap of matrix of GO enrichment scores using GSVA algorithm. **C** Volcano map showed DEGs between two patterns. **D** KEGG enrichment analysis of DEGs. *p < 0.05; **p < 0.01; ***p < 0.001; *ns* no statistical significance

As hypothesized, the analysis of methylation modification patterns, as assessed through MRGs, revealed that subtype A patients, where rupture occurred, were predominant compared to subtype B, where rupture did not occur. To further elucidate the underlying biological features contributing to distinct methylation modification patterns, we compared the differences in pathway activity between the two subtypes using gene set variation analysis (GSVA). KEGG pathway analysis showed that the TGF BETA SIGNALING PATHWAY, VASOPRESSIN REGULATED WATER REABSORPTION, SELENOAMINO ACID METABOLISM, and RNA DEGRADATION pathways were significantly upregulated in subtype A compared to subtype B (Fig. 4A). And in terms of biological function, various protein modification-related processes are significantly activated in isoform A, such as PROTEIN MONOUBIQUITINATION, HISTONE H2A MONOUBIQUITINATION, HISTONE UBIQUITINATION, PROTEIN SUMOYLATION (Fig. 4B). These findings provide insights into the underlying biological mechanisms of IA pathogenesis and warrant further investigation. The present study utilized the limma package to identify 1735 differential genes in distinct isoforms (Fig. 4C). Subsequently, an enriched pathway analysis was conducted, revealing a potential involvement of the PI3K-Akt signaling pathway in IA rupture (Fig. 4D). The findings emphasize the relevance of molecular mechanisms underlying IA pathogenesis and provide a basis for further investigations towards targeted therapeutic interventions.

In summary, our investigation identified significant alterations in the protein modification status of the A isoform, in addition to the previously reported changes in its methylation level, which may contribute to the pathogenesis of IA rupture. Moreover, our functional analysis supports the involvement of the TGF BETA SIGNALING PATHWAY and the PI3K-Akt signaling pathway in this phenomenon. These findings provide novel insights into the molecular mechanisms underlying IA pathology and may facilitate the development of targeted therapeutic interventions.

### Single cell level expression of MRGs

**Fig. 5:**
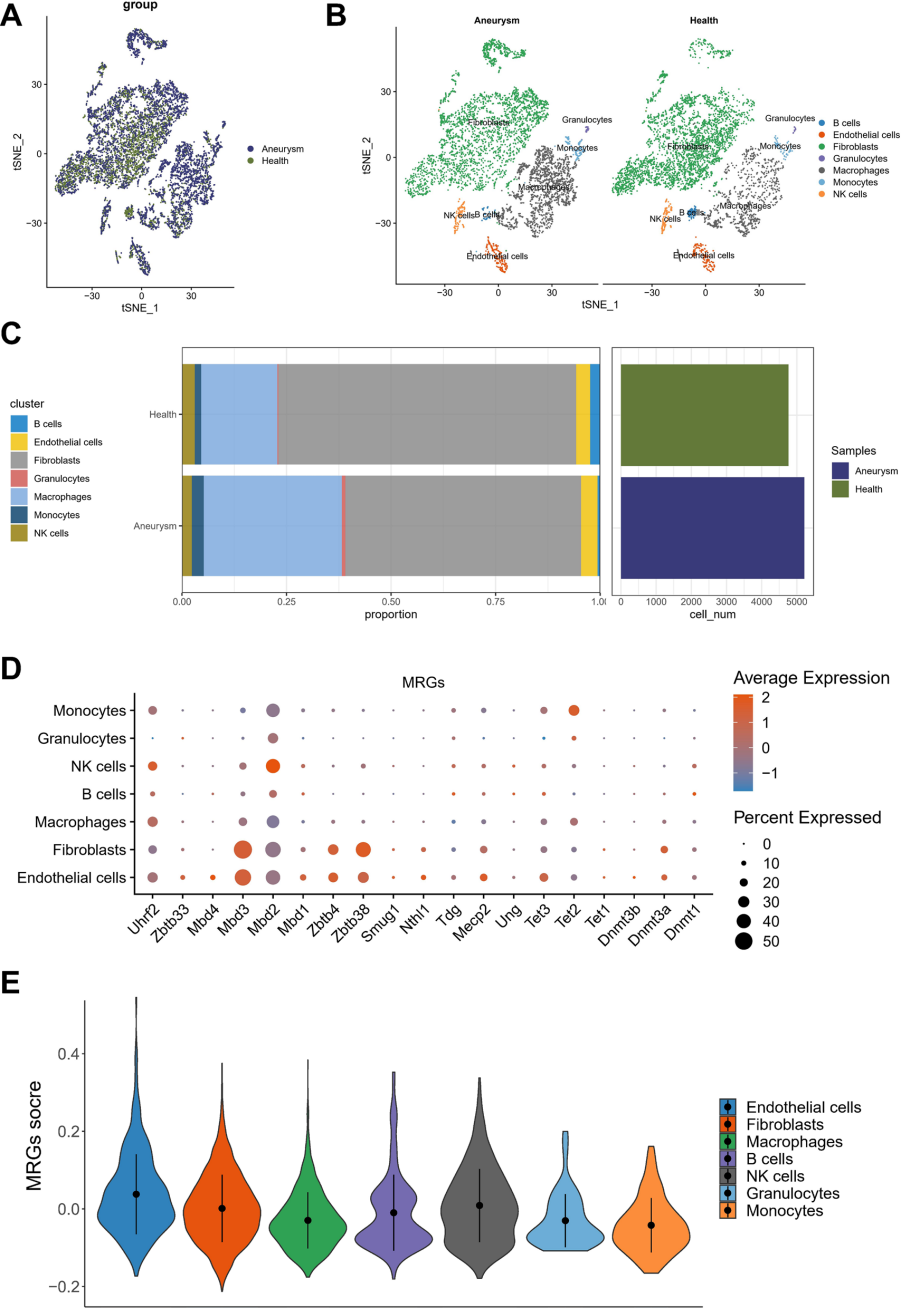
Single-cell RNA-sequencing analysis identifies Aneurysm and Health cell marker genes. **A** T-SNE plots show cells from Aneurysm and Health samples. **B** The cell types identified by marker genes. **C** The proportion Plot The cell types identified by marker genes. **D**, **E** The UMAP and boxplot plots represent 7 cell clusters from Aneurysm and Health samples

Upon integration of IA and normal samples using Seurat's CAA algorithm and preliminary identification of cell clusters, a tsne plot revealed improved sample merging (Fig. 5A). By employing the singleR package and CellMarker database to assess expression of specific markers, we successfully identified 20 distinguishable cell clusters, including fibroblasts, macrophages, NK cells, endothelial cells, B cells, granulocytes, and monocytes (Fig. 5B). Subsequent quantification of cell type proportions in IA and normal tissues indicated a predominance of fibroblasts and macrophages, with a significant increase in macrophages following IA onset and a considerable loss of fibroblast content, representing vascular composition (Fig. 5C). Analysis of differentially expressed MRGs across various cells unveiled 19 candidates, including Mdb3 and Zbtb38, which exhibited high expression in endothelial cells and fibroblasts (Fig. 5D). Overall MRG scores were evaluated without accounting for cell types and yielded the highest levels in endothelial cells (Fig. 5E).

**Fig. 6:**
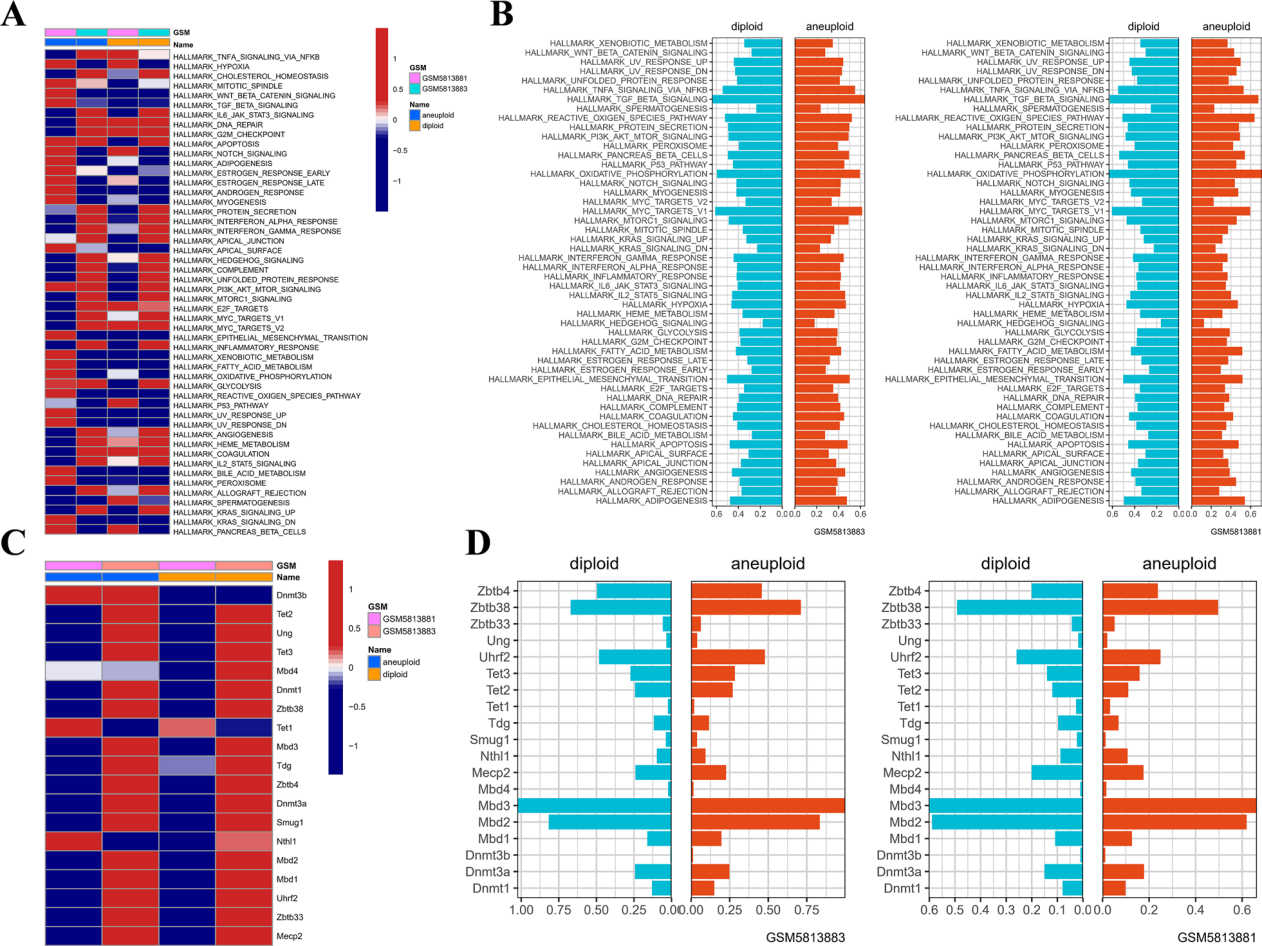
The role of DNA methylation in IA and normal cells. **A** Difference in activation of HALLMARK pathways between IA and normal cells. **B** The enrichment scores of different HALLMARK signal pathways in normal and IA cells of each Intracranial Aneurysm sample. **C** Difference in expression of DNA methylation-related genes between IA and normal cells. **D** The expression of DNA methylation-related genes in normal and IA cells of each Intracranial aneurysm sample

After segregating IA cells from normal ones, ssGSEA was employed to determine the enrichment scores of HALLMARK and MRGs pathways in individual cells. Our analyses divulged that MRGs exhibited a noteworthy decrement in IA cells relative to normal cells (Fig. 6A, B). Likewise, the gene expression levels of majority of MRGs were found to be diminished in IA cells when compared to normal cells (Fig. 6C, D).

The observations presented here provide compelling evidence that DNA methylation status in endothelial cells is a critical factor underlying the pathogenesis of IA, while such an association is not evident in immune cells. These findings may have far-reaching implications for the identification of novel molecular targets and therapeutic strategies for tackling IA, with an emphasis on modulating the DNA methylation patterns in endothelial cells.

### MRGs can be used as a potential diagnostic marker to identify rupture in patients with UIA

**Fig. 7:**
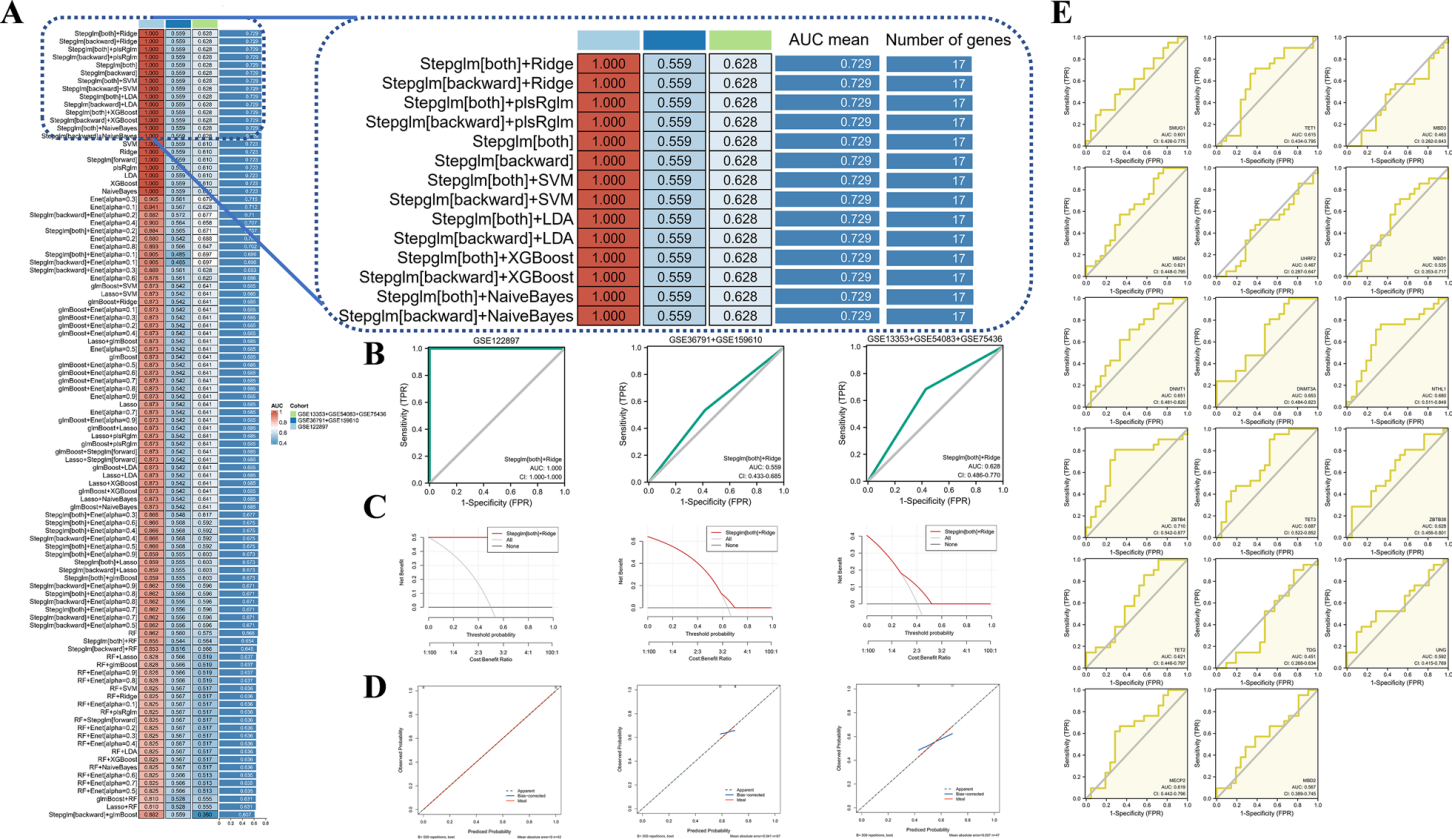
Construction and testing of the Methylation-related genes (MRGs) riskscore. **A** The AUC value of 100 machine-learning algorithm combinations in the three testing cohorts. ROC curves (**B**), decision curves (**C**) and calibration curves (**D**) were used in MRGs to identify RIA and uIA in the GSE122897 cohort, GSE36791 + GSE159610 cohort and GSE13353 + GSE54083 + GSE75436 cohort. **E** Diagnostic value of 17 diagnostic genes in MRGs

**Fig. 8:**
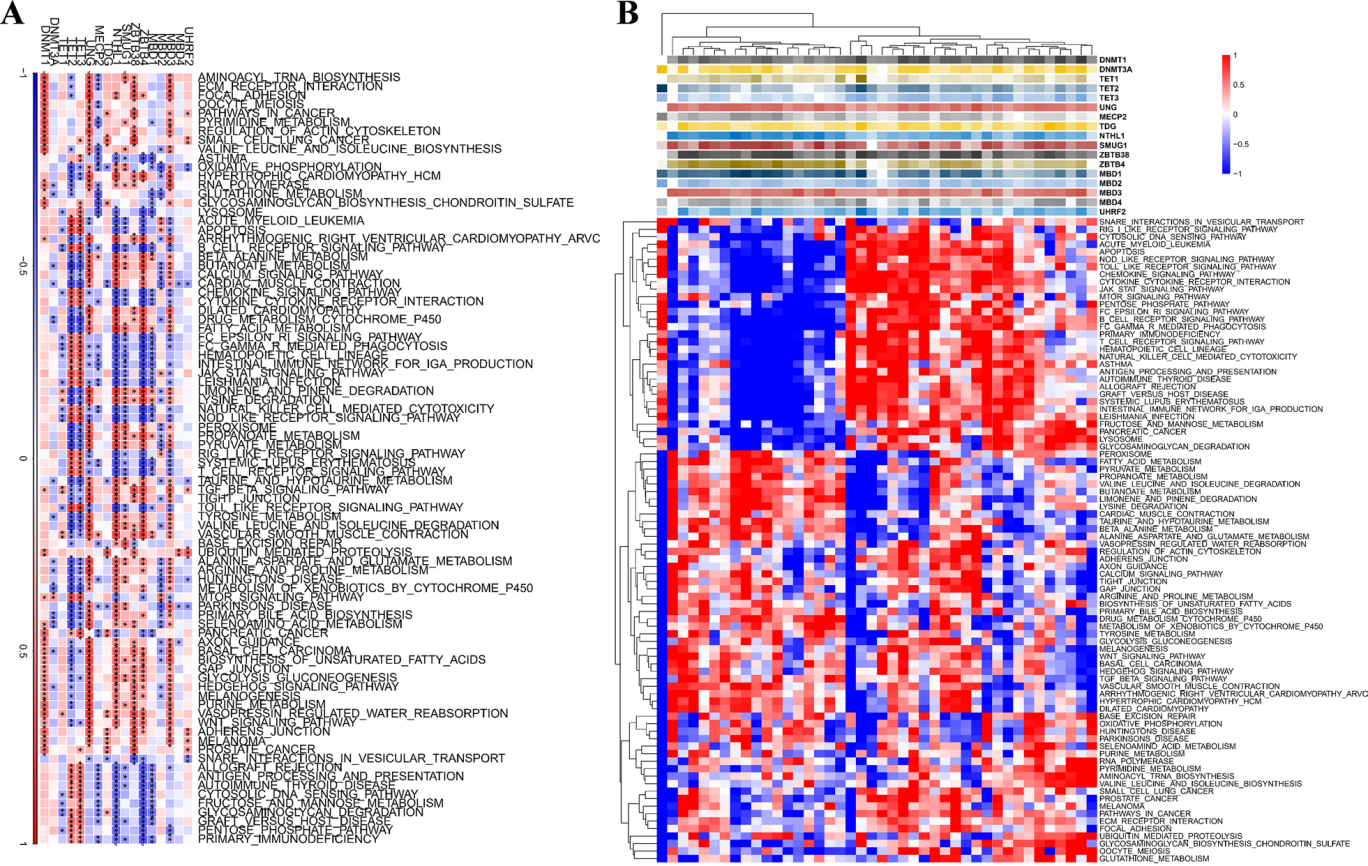
Identification of pathways that the 17 risk genes involved in. **A** Gene-pathway correlation heatmap; **B** Enrichment score heatmap for key pathways. *p < 0.05; **p < 0.01; ***p < 0.001; *ns* no statistical significance

We will develop a comprehensive prognostic model using a combination of 100 final machine learning methods to determine whether these 19 MRGs can construct a cross-organizational, comprehensive, predictive model for predicting IA rupture. Finally, the AUCs of 100 machine learning models in different cohorts were averaged using GSE122897 as the training set and other datasets as the validation set. Interestingly, 14 models showed excellent average accuracy (1.00), but we found that the Stepglm[both] + Ridge algorithm had the best model performance efficacy (Fig. 7A). In addition, the model performance was also evaluated by the ROC curve, Calibration curve and DCA curve. The MRGs model identified AUC equal to 1.00 (95% CI: 1.00–1.00) for the GSE122897 cohort, 0.559 (95% CI: 0.433–0.658) for the GSE36791 + GSE159610 cohort, and 0.628 (95% CI: 0.486–0.770) for the GSE13353 + GSE54083 + GSE75436 cohort, which showed good discrimination (Fig. 7B). The DCA suggests that MRGs models demonstrate a better clinical net benefit than either complete treatment or no treatment strategy (Fig. 7C). Calibration curves showed high agreement between model predictions and actual observed values, with a Hosmer–Lemeshow goodness-of-fit test P = 1.00, indicating that the performance of the MRGs model in the GSE122897 cohort was considerable (Fig. 7D). These data suggest that the MRGs model adequately detects patients with and without IA rupture and can effectively optimize the clinical decision-making process in IA patients. To understand the diagnostic value of the 17 genes, we plotted the ROC curve in the GSE122897 cohort and the results indicated an excellent diagnostic efficacy (Fig. 7E). We present heatmap and box plots for 17 genes in the rIA and uIA groups in each of the three cohorts (Additional file 3: Fig. S3A–C). In addition, we analyzed potential pathways associated with these 17 risk genes. As shown in Fig. 8A, B, 187 pathways were significantly associated with these six genes, including metabolism, DNA repair mode, genetic factor signaling pathway, and apoptosis.

### Correlation analysis of the MRGs score and immune regulation

**Fig. 9:**
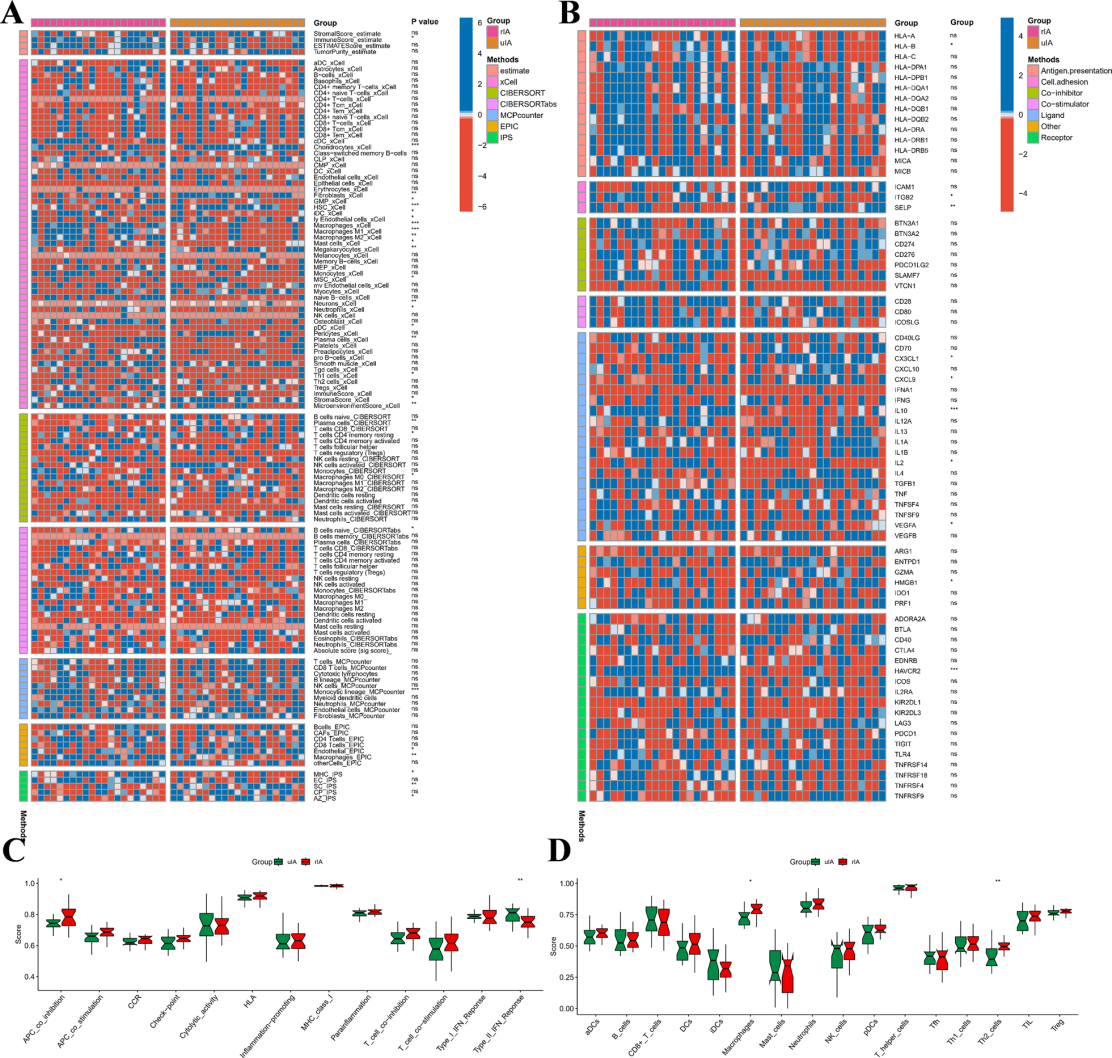
The difference in immune infiltration among patients in RIA and UIA. **A** Heat map showing differences in immune infiltrating cells between RIA and UIA. **B** Heat map showing molecular differences in immunomodulators between RIA and UIA. **C**, **D** The box plot illustrated the absolute abundance scores of the 16 immune cells and 13 immune function components in UIA and RIA. *p < 0.05; **p < 0.01; ***p < 0.001; *ns* no statistical significance

In order to further elucidate the impact of the proposed MRGs score on shaping the immune microenvironment, we utilized seven immune deconvolution algorithms comprising IPS, EPIC, MCPcounter, CIBERSORT, CIBERSORTabs, xCELL, and ESTIMATE to ensure the robustness and reproducibility of our findings and to depict the immune landscapes of the UIA and RIA groups as depicted in Fig. 9A. Comparative analysis of immune cells and functional pathways provided evidence for the existence of distinct immune cell subsets between the uIA and rIA groups related to APC co-stimulation such as checkpoint, HLA molecules, MHC class I, T cell co-inhibition, T cell co-stimulation, and type I and type II IFN responses, in addition to B cells, iDCs, mast cells, neutrophils, NK cells, T helper cells, Th1, Th2, and Treg (Fig. 9B). We also scrutinized whether immune checkpoint inhibitors were regulated in response to the MRGs scores, and noted no significant differences in the expression of most immunological checkpoints between the UIA and RIA groups (Fig. 9C), which was also not statistically significant in B cells, iDCs, mast cells, neutrophils, NK cells, T helper cells, Tfh, Th1, TIL and Treg, etc. (Fig. 9D). These results suggest that the MRGs scores may not be related to immuno-therapy.

### Establishment of a 17 MRGs genes-based nomogram for predicting intracranial aneurysm progression

An innovative diagnostic tool for the progression of Intracranial Aneurysm has been developed by integrating 17 MRGs genes and clinical features, resulting in the construction of a nomogram (Additional file 4: Fig. S4A). This nomogram assigns a score to each of the MRGs genes, with the total score being calculated by summing up the score of all 17 MRGs genes. Accordingly, different scores correspond to varying levels of risk for the progression of Intracranial Aneurysm. Calibration curves demonstrate the accuracy of this nomogram in the estimation of Intracranial Aneurysm progression. Moreover, decision curve analysis validates the clinical benefit of this novel nomogram for patients with Intracranial Aneurysm (Additional file 4: Fig. S4B). These findings hold significant implications for the early diagnosis and prompt treatment of IA (Additional file 4: Fig. S4C).

### Analysis and prediction of key miRNAs and TFs

The present study utilized an online database to predict upstream transcription factors (TFs) and miRNA-targeted diagnostic genes. The interaction network revealed 17 diagnostic genes in conjunction with 487 TFs (Additional file 5: Fig. S5A) and 763 miRNA gene regulatory networks (Additional file 5: Fig. S5B) illustrates the interaction network of the aforementioned entities. The results presented herein provide new insights into potential diagnostic targets for further investigation in the context of related diseases.

### GWAS analysis

**Fig. 10:**
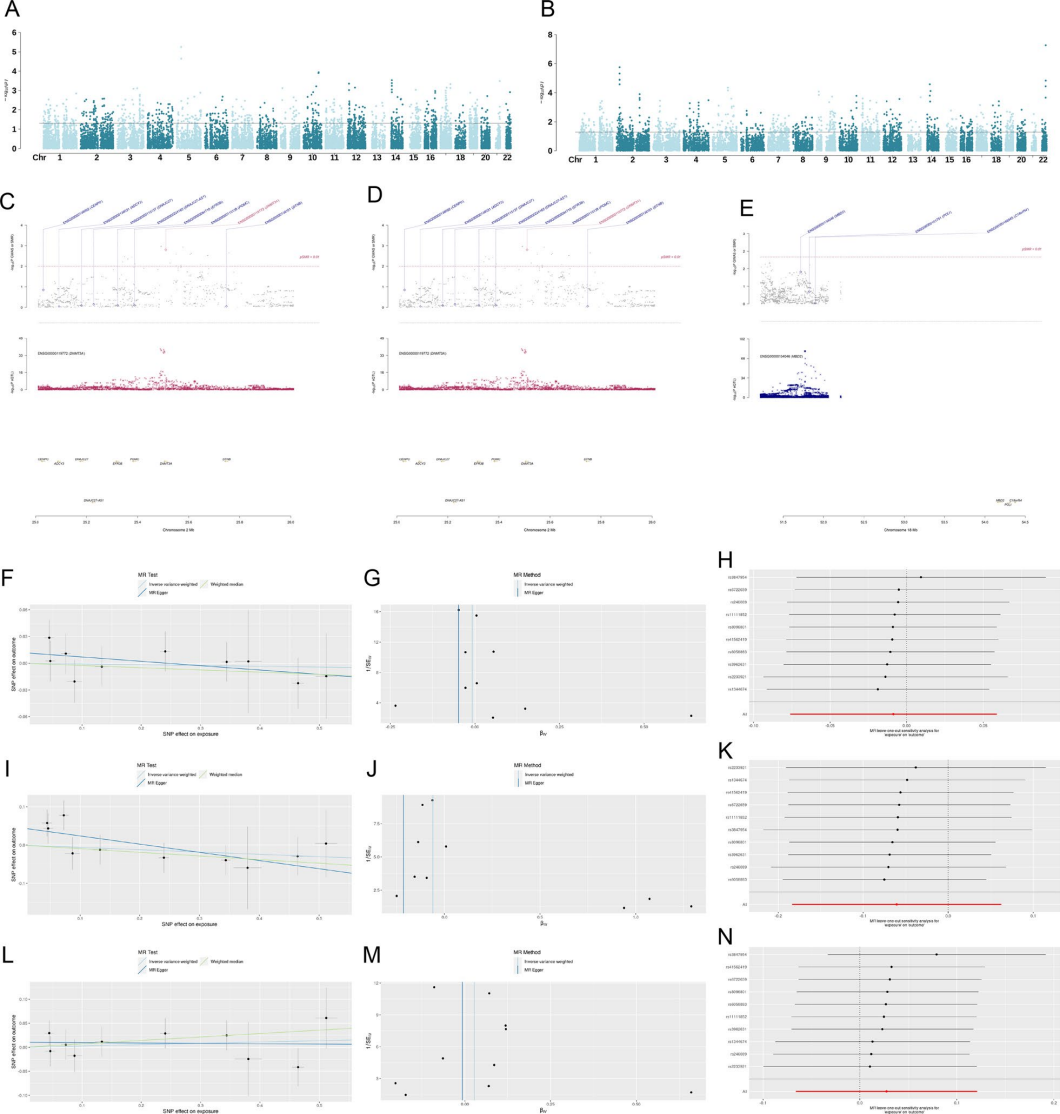
**A**, **B** Manhattan plot shows GWAS results. The y-axis indicates the Z-score for each gene tested on all autosomal and single nucleotide polymorphism weight sets. The x-axis indicates the chromosomal position corresponding to the gene, and the black line indicates the threshold of significance. **C**–**E** Pleiotropic association of DNMT3A with SAH/UIA (**C**, **D**) and MBD2 with UIA (**E**) using genome-wide cis-eQTLs. Top plot, grey dots represent the – log10 (p values) for SNPs from the GWAS of SAH/UIA, with solid rhombuses indicating that the probes pass HEIDI test. Middle plot, eQTL results. Bottom plot, location of genes tagged by the probes. Effect estimates IA, SAH, and UIA (**F**, **N**). Investigation of the association of a genetically determined unit increase in exposure with the risk of IA/SAH/UIA using inverse-variance weighted, MR Egger, and weighted median estimates. **F**, **I**, **L** Scatter plots of individual SNP effects and estimates from different MR techniques for the effect of DNA methylation related-genes on IA/SAH/UIA. **G**, **J**, **M**, Funnel plots of DNA methylation related-genes on IA/SAH/UIA. **H**, **K**, **N** Leave-one-out analysis plots for DNA methylation related-genes on IA/SAH/UIA. *eQTL* expression quantitative trait loci, *GWAS* genome–wide association studies, *HEIDI* heterogeneity in dependent instruments, *SMR* summary data–based Mendelian randomization, *SNP* single nucleotide polymorphism, *SAH* subarachnoid hemorrhage, *IA* Intracranial aneurysm

Through gene set analysis of DNA methylation regulators, our investigation uncovered the presence of the ZBTB4 and DNMT3A genes in individuals with SAH, displaying p-values of 0.024137 and 0.041593, respectively. Notably, a DNMT3A gene was also detected in patients with uIA disease, demonstrating a p-value of 0.012629. These findings are fully documented in Additional file 6 and are visually represented in Fig. 10A, B.

### SMR analysis of genome-wide cis-eQTLs and IA/aSAH/UIA

To identify genetic loci associated with SAH and UIA, we performed a pleiotropy-robust analysis using P-SMR < 0.05 and P-HEIDI > 0.01. With these stringent criteria, we identified one association signal for a unique genetic locus associated with SAH and two association signals for two unique genetic loci in uIA. Specifically, we found a significant association between DNMT3A and a reduced risk of both SAH (OR = 0.41, 95% CI = − 2.1 to 3.01, Psmr = 0.00156) and uIA (OR = 0.36, 95% CI = − 2.52 to 3.25. Psmr = 0.014), while MBD2 was found to significantly increase the risk of uIA (OR = 1.68, 95% CI = − 0.85 to 4.21, Psmr = 0.043). Unfortunately, we did not observe any association signal for IA. Our findings suggest that DNMT3A and MBD2 may play important roles in the pathogenesis of SAH and uIA, respectively (see Additional file 7: Table S1 and Fig. 10C–E for details)

### SMR analysis of genome-wide cis-mQTLs and IA/SAH/UIA

Following rigorous quality control measures, a conventional SMR analysis was executed, though no discernible causal associations were identified. A plausible factor contributing to this outcome is the limited scope of gene selection, alongside the notion that methylation levels altering the selected genes may not have a considerable impact on the genetic variation.

### MR analysis of genome-wide cis-eQTLs and IA/SAH/UIA

In our study, we initially conducted a two-sample MR analysis for IA, SAH, and UIA with 19 associated genes using the TwoSampleMR framework. Unfortunately, we did not find any causal associations with IA, SAH, or UIA in the overall analysis (Fig. 10F–N). Subsequently, we performed a two-sample MR analysis for each of the 19 genes corresponding to specific SNPs with IA, SAH, and UIA. We observed that DNMT1 played a significant protective role in SAH (OR = 0.28, 95% CI = 0.09 to 0.88, Pivw = 0.029), while DNMT3B was identified as a risk factor for uIA (OR = 2.92, 95% CI = 1.01 to 8.44, Pivw = 0.011) (Additional file 8: Table S2). To further investigate the relationship between the selected genes and IA, SAH, and UIA, we employed multiple MR methodologies with the aim of validating and functionally annotating these causal connections. This approach was conducted in comparison with previous transcriptome-based analyses.

## Discussion

DNA methylation, an epigenetic modification, has attracted significant attention due to its association with a wide array of diseases. This has led to the development of various therapeutic strategies, such as the use of histone deacetylase inhibitors and DNA methyltransferase inhibitors [48]. The disruption of DNA methylation patterns has been observed in numerous diseases, including cancer, cardiovascular disorders, and neurological conditions. Consequently, further investigation of this epigenetic phenomenon and its role in disease pathogenesis is essential for advancing our understanding and the development of therapeutic approaches [49]. The importance of investigating the diagnostic value and underlying mechanisms of DNA methylation-related enzymes in IA cannot be overstated, as this could lead to more effective treatments and improved prognoses for IA patients. In our study, we utilized machine learning-based approaches to develop risk indicators using expression profiles of DNA methylation-related genes. We fitted 100 binary models in a training set and subsequently replicated them in a combined training set cohort (GSE13353, GSE54083, and GSE75436) and two external validation cohorts (GSE36791 & GSE159610 cohort and GSE122897 cohort). We selected the Stepglm [both] + Ridge algorithm due to its high average accuracy, low model gene number, and optimal model performance power. The combined application of multiple machine learning algorithms enables more efficient variable dimensionality reduction, thus facilitating the development of accurate and simple predictive models. Model performance was assessed using receiver operating characteristic (ROC) curves, calibration curves, and decision curve analysis (DCA) in the public cohort. All evaluation methods demonstrated the high accuracy of our DNA methylation-related gene models in identifying both UIA and RIA patients.

Progression and rupture of IAs encompass a multitude of contributing pathological factors, with epigenetic modifications, immune-inflammation, and vascular stability being of utmost importance. Recent research by Poppenberg et al. has utilized ENCODE data to explore the epigenetic landscape of 16 prominent IA risk haplotypes [50]. The researchers discovered that genetic alterations, such as SNPs, within these haplotype blocks might influence the transcription of genes implicated in IA development by modulating enhancer activity. Interestingly, functional regulatory elements within IA-associated risk regions were found to be more abundant in endothelial cells compared to immune cells. No statistically significant enrichment of histone marks or CTCF binding sites was observed in IA-associated LD blocks in neutrophils, suggesting that functional regulatory elements in IA-associated risk regions predominantly occur in endothelial cells. Thus, the genetic risk associated with IA is more likely conferred by endothelial cells rather than immune cells. Consequently, aberrant gene expression detected in circulating neutrophils may signify the presence of aneurysmal lesions instead of genetic predispositions, prior to the onset of IA [50].

Moreover, the absence of significant enrichment of histone marks or CTCF binding sites in IA-associated linkage disequilibrium blocks in neutrophils suggests that the primary contribution to the genetic risk of IA originates from endothelial cells. This observation reinforces the notion that altered gene expression detected in circulating neutrophils may not manifest genetic predispositions, but rather indicate the presence of aneurysmal lesions before the onset of IA. Furthermore, gene ontology data supports the primary role of endothelial cells in IA pathogenesis by underlining the importance of endopeptidase activity/regulation and extracellular matrix (ECM) structural components, which are likely to be more pertinent to the function of endothelial cells rather than immune cells (neutrophils) [50]. Proficiently understanding these subtle microscopic alterations may facilitate a more accurate assessment of pathological conditions and the risk of aneurysm. In this investigation, we conducted a comprehensive analysis of divergent methylation modification patterns within different intracranial aneurysm (IA) samples utilizing the CIBERSORT algorithm. Our findings revealed varying proportions of distinct immune cells associated with specific methylation modification patterns, suggesting that the activation state of methylation at the tissue level could potentially play a crucial role in IA rupture, independent of immune-related factors. In addition, we performed single-cell sequencing to compare the MRGs score in cell types without use and found that endothelial cells had the highest MRGs score levels. During the development of IA, the methylation level of endothelial cells plays a critical left and right but may not be related to changes in the methylation of immune cells. We again validate the findings of Poppenberg et al. that Epigenetic landscapes suggest that genetic risk for intracranial aneurysm operates on the endothelium.

Clinical decision-making for IA can be challenging, as the available polygenic models for UIA or RIA have been constructed using healthy subjects as controls, without in-depth assessment of molecular pathological features. Accordingly, biomarkers that identify high-risk patients from populations with established IA may provide more clinically-relevant information. Thus, the aim of this study is to identify genetic biomarkers that can distinguish ruptured from UIA in both tissue and peripheral blood samples, enabling construction of more robust polygenic models for diagnostic or predictive purposes. In previous work, our group investigated the role of m6A regulator-mediated RNA methylation modification patterns [51] as well as classification patterns of immunogenic cell death-related regulators, in IA. However, in this study, we will focus on the identification of genetic biomarkers that are specific to established IA, rather than healthy controls [52].

Recent advancements in GWAS have led to the identification of numerous loci that are linked to complex diseases and traits. GWAS utilizes linkage disequilibrium correlation structure of the genome to inexpensively capture alterations by genotyping a large number of variants and extrapolating the genotypes of non-genotyped variants with a compact genotyping reference panel [53]. However, since LD correlations often point towards genomic regions containing numerous genes, the prioritization of functionally-relevant genes solely with GWAS data can pose a significant challenge. This renders laboratory-based tracking of related regions in search of possible pathogenic variants a costlier and less practical option.

Despite the identification of risk loci for IA, there remains a lack of comprehensive explanations for the heritability of this condition. However, previous studies utilizing integration of data from GWAS and eQTL have proven successful in enhancing the discovery of risk loci and providing biological insights into their function. GWAS analysis has revealed a significant association between the risk of SAH and genes ZBTB4 and DNMT3A, while UIA only harbors a single DNMT3A gene. ZBTB4 has been implicated in a multifaceted range of functions, including DNA-binding transcriptional blocking activity, methyl-CpNpG binding activity, sequence-specific DNA binding activity, and RNA polymerase II specificity. It is associated with the cellular response to DNA damage stimuli and negative regulation of RNA polymerase II transcription. Recently, the role of METTL3-mediated RNA methylation of m6ZBTB4 in trophoblastic invasion and recurrent spontaneous abortion (RSA) has been highlighted [54]. In addition, METTL3-mediated modification of m6ZBTB4 mRNA is also involved in smoking-induced EMT in lung cancer [55], Recent findings demonstrate a role for ZBTB4 in epigenetic regulation, particularly in DNA methylation, and its dysfunction has been implicated in recurrent spontaneous abortion and smoking-induced epithelial-to-mesenchymal transition in lung cancer.

In this study, we conducted SMR analysis to identify new causal genes associated with IA and investigate their functional significance. Our analysis identified one association signal for one unique genetic locus associated with SAH and two association signals for two unique genetic loci in UIA. Notably, we observed a significant association between DNMT3A and a reduced risk of both SAH and UIA, while MBD2 was associated with an increased risk of UIA, indicating the importance of DNA methylation in IA pathogenesis. The findings of the present study offer insights into the pathogenesis of IA, particularly with respect to the contribution of DNA methylation. The identification of DNMT3A and MBD2 as causal genes associated with IA is noteworthy in light of their established roles in DNA methylation. Specifically, DNMT3A is a de novo methyltransferase that catalyzes the methylation of unmethylated cytosines in CpG dinucleotides to establish DNA methylation patterns. While DNMT3A mutations have been reported in hematologic malignancies, recent studies have linked DNMT3A mutations to developmental growth disorders, such as Tatton-Brown-Rachman syndrome and microcephaly dwarfism. In mice, DNMT3A has been shown to be indispensable for postnatal development and is involved in multiple processes in the nervous system. The observed association between DNMT3A and a reduced risk of intracranial aneurysm is thus of particular significance, suggesting that DNA methylation, in part mediated by DNMT3A, may play a critical role in IA pathogenesis. Conversely, MBD2 was found to be associated with an increased risk of unruptured intracranial aneurysm, underscoring the complex nature of the epigenetic mechanisms underlying IA. These findings contribute to a deeper understanding of IA etiology and may offer potential targets for future therapeutic interventions [56].

DNMT3A contains a C-terminal catalytic methyltransferase structural domain and two known regulatory structural domains [57]. ATRX-DNMT3-DNMT3L and Pro-Trp-Trp-Pro (PWWP). The ATRX-DNMT3-DNMT3L domain engages with the unmethylated lysine 4 (H3K4me0) of histone H3, directing methylation away from the active promoter. In contrast, the PWWP domain is thought to recognize various histone marks, including H3K36me3 and H3K36me2, facilitating efficient DNA methylation of genomic and intergenic regions [58]. The interaction between regulatory domains and histone modifications, among other factors, enables DNMT3A to affect chromatin structure and gene expression in a sophisticated and intricate manner. Notably, DNMT3A has two major protein isoforms: DNMT3A1 containing a 219 amino acid N-terminal structural domain, and DNMT3A2 transcribed from the intron promoter and spliced to the downstream exon, thereby lacking the N-terminal structural domain. DNMT3A is a key regulator of DNA methylation and plays a critical role in controlling spermatogonial stem cell plasticity [56]. Recent studies have shown that DNMT3A-dependent DNA methylation is required for spermatogonial stem cell commitment to spermatogenesis. Specifically, Dnmt3A mutant SSCs are associated with spurious enhancer activation that implements irreversible stem cell genetic programs, highlighting DNMT3A as a crucial factor in controlling stem cell fate decisions [59]. Additionally, the 2.65-Å crystal structure of the DNMT3A-DNMT3L-DNA complex has recently been elucidated, demonstrating how two DNMT3A monomers attack two CpG dinucleotides simultaneously, separated by fourteen base pairs in the same DNA duplex. Further scrutiny of DNMT3A-DNA interactions revealed the importance of the target recognition domain (TRD), catalytic ring, and DNMT3A homodimer interface in DNMT3A activity. Notably, mutations in the substrate binding residues of DNMT3A have been shown to reduce enzymatic activity, induce CpG hypermethylation, and promote hematopoietic cell transformation—revealing the etiological link between DNMT3A-mediated DNA methylation and human disease [60]. DNMT3A deletion and overexpression have been shown to impact mCA levels in a dose-dependent manner, with heterozygous deletion resulting in a 50% reduction in genome-wide mCA and moderate overexpression following microRNA regulatory deletion leading to excessive deposition of this epigenetic marker [61, 62]. The role of mCA in transcriptional regulation involves recruiting the methyl-binding protein MeCP2 to pass through enhancers and regulate the expression of genes involved in neuronal activity and maintaining cell type-specific gene expression throughout the genome [63]. The transcriptional regulation of target genes by methyl-CpG binding domain 2 (MBD2) occurs through binding to methylated CpG DNA to translate the information encoded in DNA methylomes. Research has shown that attenuation of TGF-β1, UUO and I/R treatment-induced renal fibrosis through MBD2 silencing or PT-MBD2-KO is due to MBD2 directly leading to increased expression of EGR1 and inducing hypomethylation in the promoter region [64]. Alterations in DNA methylation and MBD2 expression can influence the maintenance of Th1 program homeostasis, via the binding of MBD2 to methylated CpG DNA within the Stat1 promoter, thereby preventing autoimmunity. The induction of ectopic MBD2 expression presents an opportunity to reduce the diabetogenicity of CD4 T cells through the attenuation of T1D in NOD.scid mice. Together, these findings suggest that MBD2 can serve as a promising avenue for the development of epigenetically-based T1D therapies in clinical practice [65].

In this novel study, a MR approach was utilized for the first time, leveraging DNA methylation-related gene data within a pooled IA dataset, to profile the causality and association of IA risk. Two-sample MR of 19 genes corresponding to SNPs with IA/SAH/UIA in two sample MR was also conducted. The results indicated that DNMT1 acts as a significant protective factor in SAH, while DNMT3B was identified as a risk factor for uIA. Notably, DNMT1 is involved in fresh DNA methylation through the hemimethylated DNA mechanism, while also functioning as a component of the epigenetic machinery responsible for gene repression via promoter methylation. Beyond its catalytic activity, DNMT1 also regulates gene expression and plays a role in various processes, including cell cycle, DNA damage repair and stem cell function [66]. The present study employs a Mendelian randomization approach utilizing DNA methylation-related gene data, in conjunction with a pooled IA dataset, to evaluate the causality and association of IA risk. Incorporating two sample MR, a two-sample polygenic approach, 19 genes corresponding to SNPs with IA/SAH/UIA were assessed. Results indicated that DNMT1 was identified as a significant protective factor in SAH while DNMT3B was identified as a risk factor for UIA. Furthermore, MCP-1-induced sustained low wall shear stress and turbulence on IA endothelial cells from human umbilical vein endothelial cells led to down-regulation of DNMT1 and decreased DNA methylation levels in the AC007362 promoter [67]. Ruptured IA tissues exhibited similar results of decreased DNA methylation levels compared to unruptured IA tissues. Dnmt3a and Dnmt3b, essential for de novo methylation and embryogenesis, exhibit non-overlapping functions in development, with dnmt3b particularly required for methylation of mitotic subsatellite repeat sequences [68]. Overall, our findings provide insight into the role of DNMT1 and DNMT3B in the DNA methylation process of IA.

The current research deploys a bioinformatics approach to scrutinize the transcriptional landscape that DNA methylation carves out in IA. This thorough examination employs a broad selection of advanced methodologies, encompassing over 100 machine learning algorithms, GWAS, MR, and SMR. However, the study acknowledges certain inherent limitations stemming from the biological context, data noise and uncertainty, predictive models, causality, incomplete knowledge of biological networks, and generalizability concerns. Transcriptomics data offer a snapshot of gene expression levels in a specific context and may miss contributions from dynamic regulatory mechanisms and cell-specific expression patterns during the pathogenesis of IA. The heterogeneity of sample sources, experimental conditions, and technical artifacts may introduce data noise and uncertainty, which can obscure the accurate association between DNA methylation and IA. Predictive models utilizing machine learning algorithms require precise tuning and validation to avoid overfitting and ensure generalizability and may not account for all clinical variations that can occur. Although MR and SMR approaches can provide evidence for potential causal relationships, establishing definitive causality between DNA methylation events and IA onset or progression remains a major challenge and requires further experimental validation. The complex, interconnected nature of biological systems may lead to incomplete or biased interpretation of the results, given the unknown or poorly understood roles of many genes and pathways in IA pathogenesis. Finally, generalizability concerns arise due to population-specific genetic and epigenetic architecture, warranting further investigation in disparate cohorts.

To overcome the prevailing obstacles, future research should integrate additional "omics" data disciplines such as proteomics and metabolomics, and investigate the involvement of other epigenetic markers in IA progression. In addition, incorporating empirical validation in relevant pathological models to reinforce the reliability of identified associations and cause-and-effect relationships while augmenting bioinformatics methodology will be crucial. By building upon these fundamentals, a thorough comprehension of the molecular pathways underlying IA pathogenesis may lead to novel diagnostic and therapeutic interventions.

## Conclusion

In this study, novel bioinformatics approaches were utilized, including transcriptomics analysis, machine learning algorithms, genome-wide association studies (GWAS), Mendelian randomization (MR), and summary data-based Mendelian randomization (SMR), to investigate the association between DNA methylation and intracranial aneurysms (IA) and to reveal potential underlying causal relationships. This comprehensive bioinformatics framework enables the systematic examination of differentially methylated genes and their impact on IA development, progression, and rupture. The integration of cutting-edge analytical methods has identified several differentially methylated genes and pathways that may be associated with IA susceptibility. Furthermore, the use of MR and SMR approaches has provided evidence supporting potential causality between the identified DNA methylation events and IA susceptibility. These findings not only enhance our understanding of the molecular mechanisms underlying IA but also highlight possible novel biomarkers and therapeutic targets for the prevention, diagnosis, and treatment of this critical cerebrovascular disorder.

### Supplementary Information

**Additional file 1: Figure S1**. Immune characteristics of the tow clusters in the IA dataset. (A) Sankey plots show the relationship between our two clusters (clusters A and B) and histological classification (normal, RIA and UIA) and sex (male and female). (B) Stacked diagram showing the components of 22 immune cell infiltrations. (C) Box plot showing 22 immune cell differentially infiltrations in A and B cluster. *p < 0.05; **p < 0.01; ***p < 0.001; ns, no statistical significance.

**Additional file 2: Figure S2**. The difference in immune infiltration among patients in A and B cluster. (A) Heat map showing differences in immune infiltrating cells between A and B cluster. (B) Heat map showing molecular differences in immunomodulators between A and B cluster. (C-D) The box plot illustrated the absolute abundance scores of the 16 immune cells and 13 immune function components in A and B cluster. *p < 0.05; **p < 0.01; ***p < 0.001; ns, no statistical significance.

**Additional file 3: Figure S3**. 19 MRGs differentially expressed in RIA and UIA. Box plots and heat maps showing 19 MRGs differentially expressed in the RIA and UIA in (A) GSE122897 cohort, (B) GSE36791 + GSE159610 combined cohort and (C) GSE13353 + GSE54083 + GSE75436 combined cohort, respectively.

**Additional file 4: Figure S4**. Create a UIA rupture risk nomogram and assess its clinical performance and benefits. (A) UIA rupture risk nomogram. Calibration curves (B) and DCA curves (C) are used to evaluate the effectiveness of the nomogram.

**Additional file 5: Figure S5**. Regulation of miRNA networks of central genes and TF networks. (a) miRNA networks regulating central genes. (b) TF network regulating central genes.

**Additional file 6.** GWAS results in IA, uIA and SAH.

**Additional file 7: Table S1.** SMR analysis of genome-wide cis-eQTLs and uIA/SAH.

**Additional file 8: Table S2**. SNP heritability estimates.

## Data Availability

The article/Supplementary material contains the original contributions made to the study. Corresponding authors can be contacted for more information. The code and procedure files are available from the following: https://github.com/soochow-Feng/DNA-Methylation-regulator.

## Abbreviations

IA: Intracranial aneurysm
UIA: Unruptured intracranial aneurysm
RIA: Ruptured intracranial aneurysm
SAH: Subarachnoid hemorrhage
EWAS: Epigenomic association studies
CpGs: Cytosine-phosphate-guanine site
mQTLs: Methylation quantitative trait loci
GWAS: Genome-wide association studies
SHBG: Sex hormone binding globulin
BioT: Bioavailable testosterone
S-Ca: Serum calcium
aSAH: Aneurysmal subarachnoid hemorrhage
MRGs: Methylation-related genes
DMRs: Differentially methylated regions
PC: Principal components
GSVA: Gene set variation analysis
ROC: Receiver operating characteristic
DCA: Decision curve analysis
ECM: Extracellular matrix
