# Supplementary figures and images for: DNA methylation regulator-mediated modification patterns and risk of intracranial aneurysm: a multi-omics and epigenome-wide association study integrating machine learning, Mendelian randomization, eQTL and mQTL data
Source: J Transl Med. 2023 Sep 23;21:660. doi: 10.1186/s12967-023-04512-w (PMC10518114; doi:10.1186/s12967-023-04512-w)

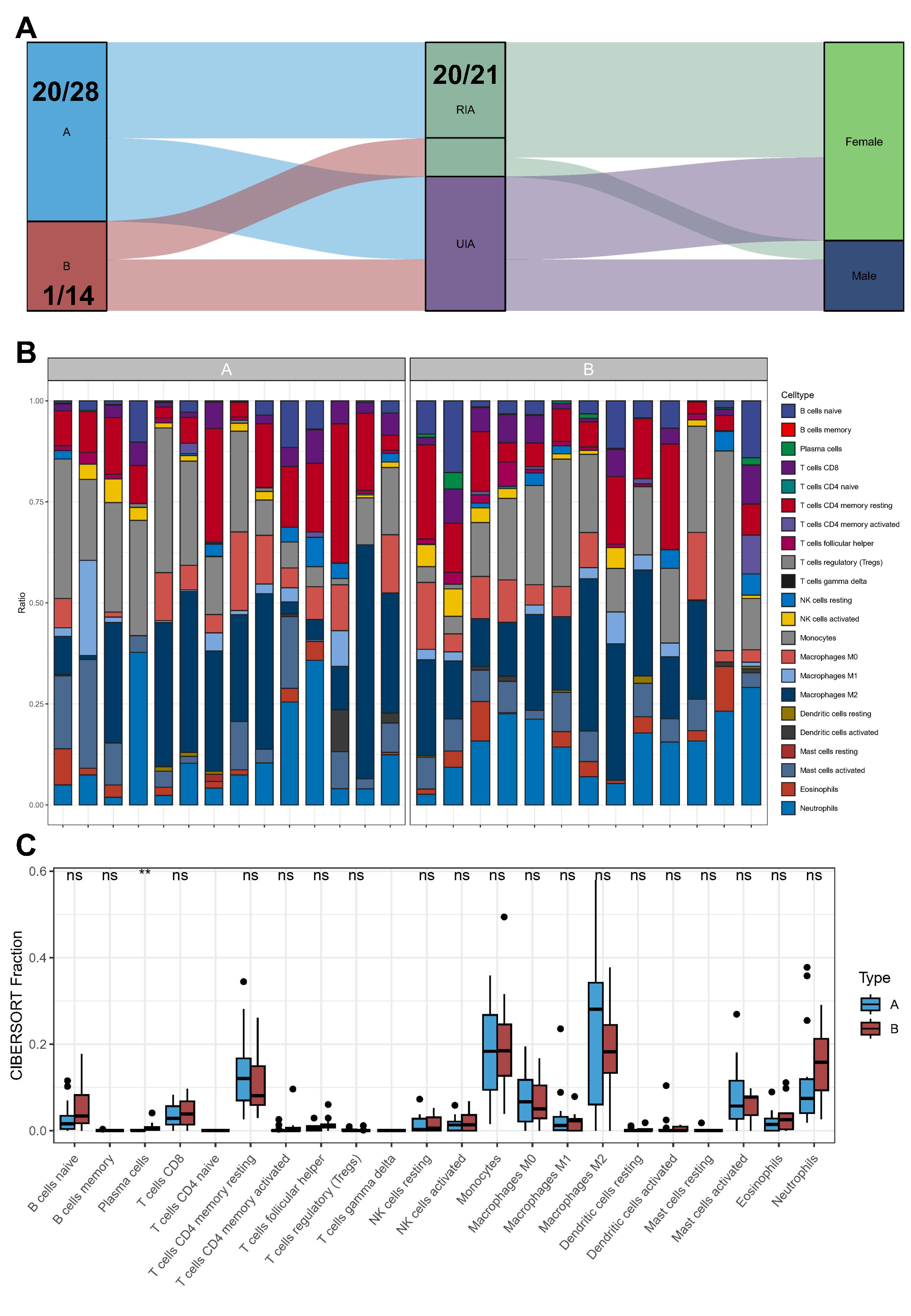

Supplement: Supplementary file 1 — Additional file 1: Figure S1. Immune characteristics of the tow clusters in the IA dataset. (A) Sankey plots show the relationship between our two clusters (clusters A and B) and histological classification (normal, RIA and UIA) and sex (male and female). (B) Stacked diagram showing the components of 22 immune cell infiltrations. (C) Box plot showing 22 immune cell differentially infiltrations in A and B cluster. *p < 0.05; **p < 0.01; ***p < 0.001; ns, no statistical significance. [file 12967_2023_4512_MOESM1_ESM.tif]

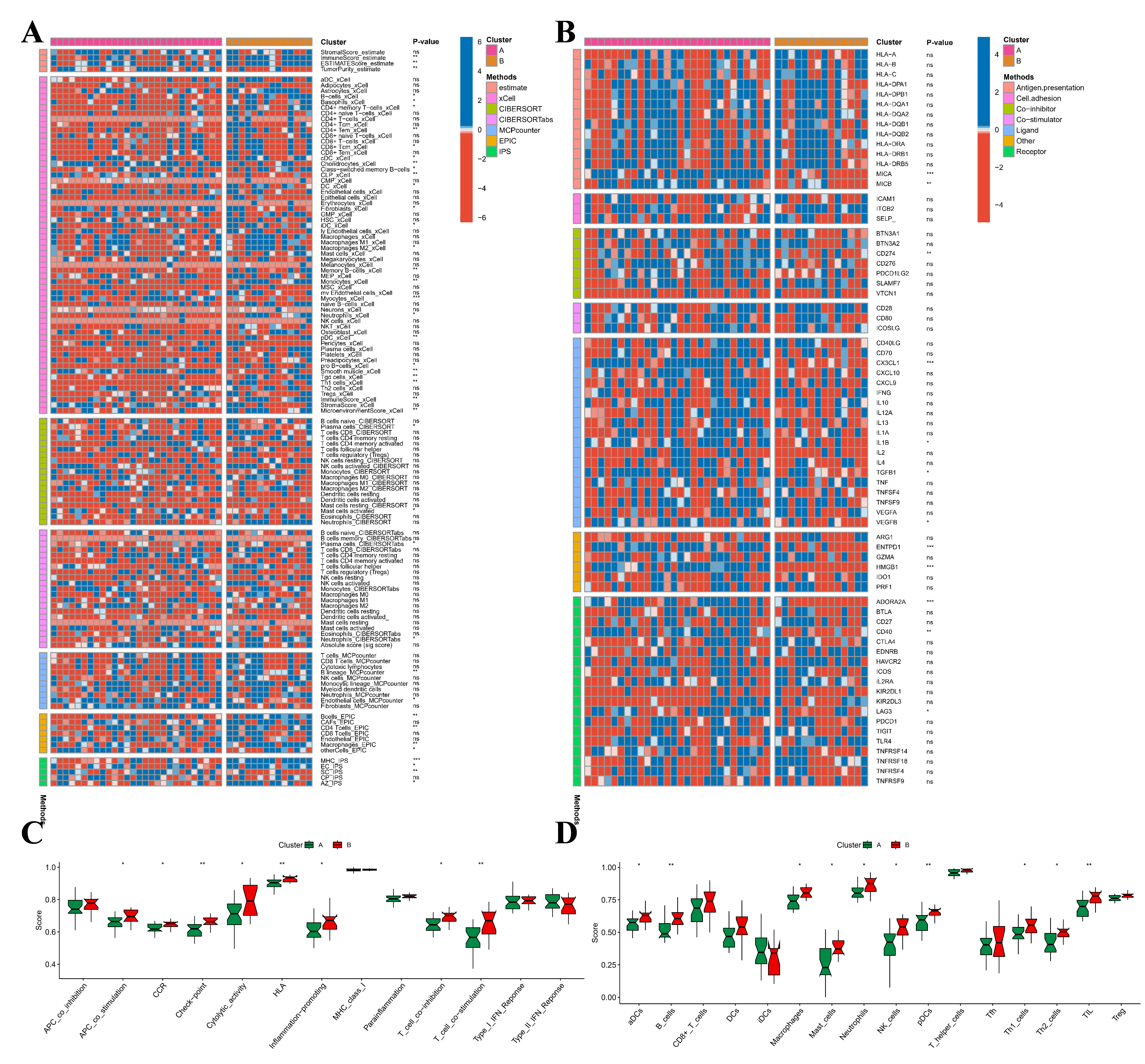

Supplement: Supplementary file 2 — Additional file 2: Figure S2. The difference in immune infiltration among patients in A and B cluster. (A) Heat map showing differences in immune infiltrating cells between A and B cluster. (B) Heat map showing molecular differences in immunomodulators between A and B cluster. (C-D) The box plot illustrated the absolute abundance scores of the 16 immune cells and 13 immune function components in A and B cluster. *p < 0.05; **p < 0.01; ***p < 0.001; ns, no statistical significance. [file 12967_2023_4512_MOESM2_ESM.tif]

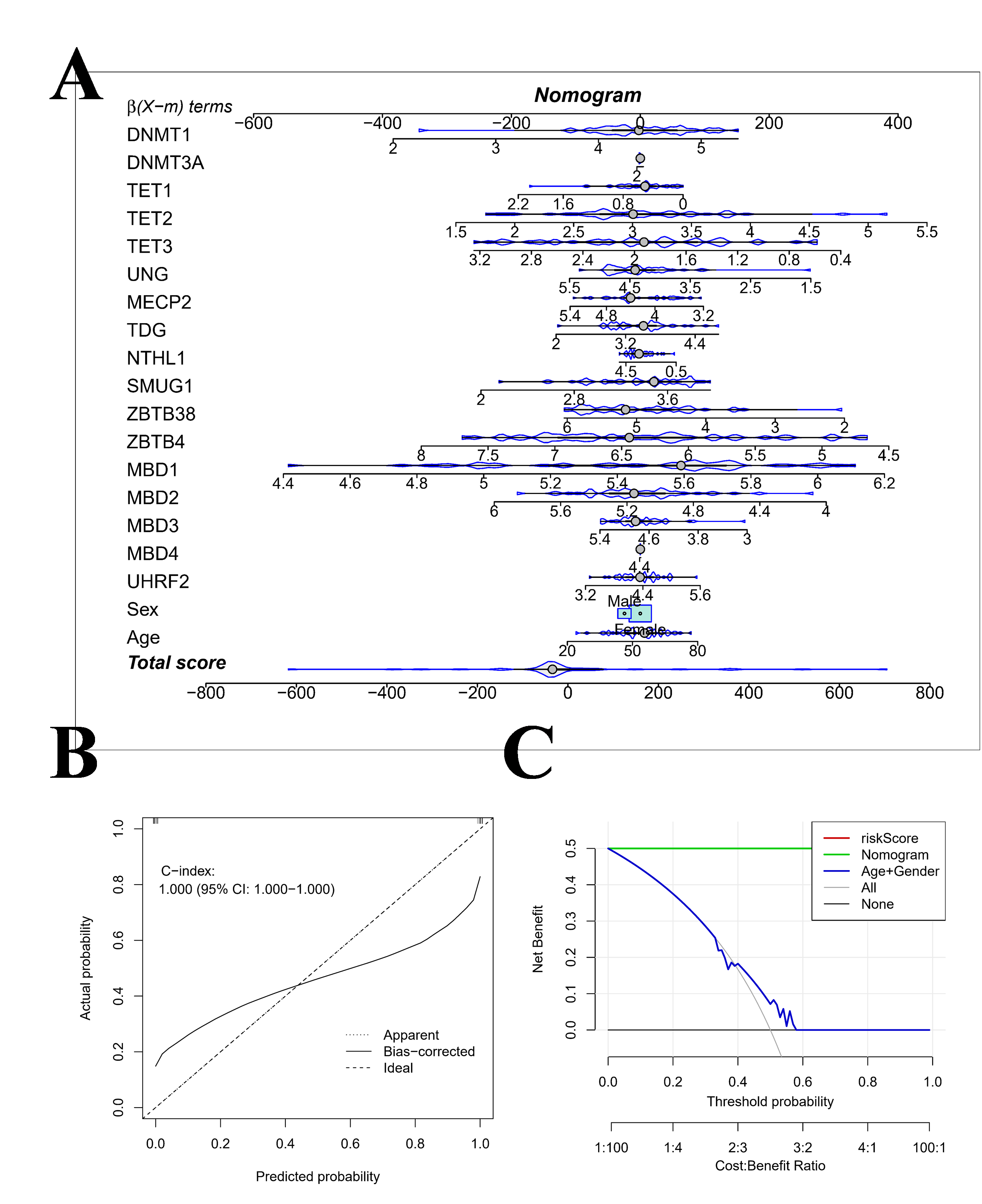

Supplement: Supplementary file 4 — Additional file 4: Figure S4. Create a UIA rupture risk nomogram and assess its clinical performance and benefits. (A) UIA rupture risk nomogram. Calibration curves (B) and DCA curves (C) are used to evaluate the effectiveness of the nomogram. [file 12967_2023_4512_MOESM4_ESM.tif]

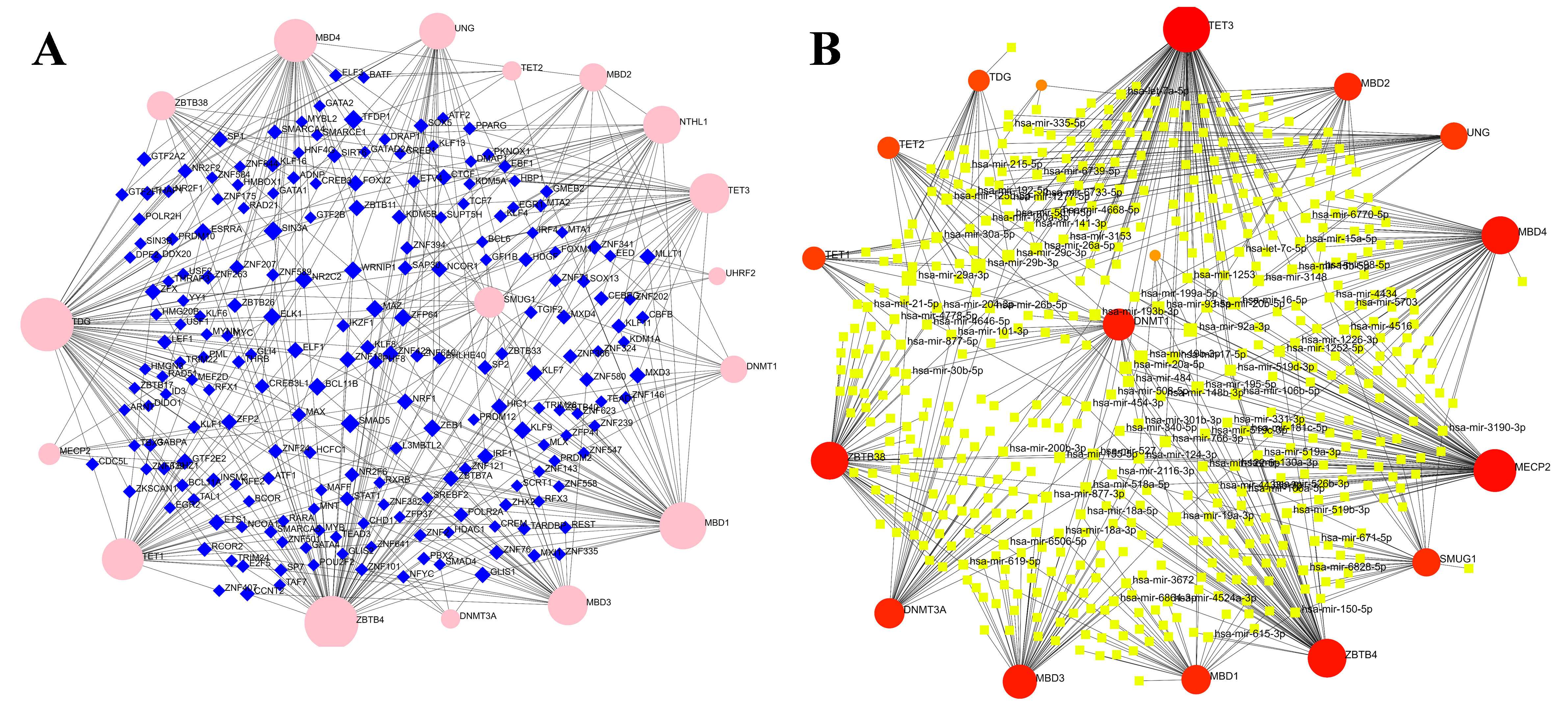

Supplement: Supplementary file 5 — Additional file 5: Figure S5. Regulation of miRNA networks of central genes and TF networks. (a) miRNA networks regulating central genes. (b) TF network regulating central genes. [file 12967_2023_4512_MOESM5_ESM.tif]
