# Supplementary material for: DNA methylation regulator-mediated modification patterns and risk of intracranial aneurysm: a multi-omics and epigenome-wide association study integrating machine learning, Mendelian randomization, eQTL and mQTL data
Source: J Transl Med. 2023 Sep 23;21:660. doi: 10.1186/s12967-023-04512-w (PMC10518114; doi:10.1186/s12967-023-04512-w)
Supplement: Supplementary file 7 — Additional file 7: Table S1. SMR analysis of genome-wide cis-eQTLs and uIA/SAH. [file 12967_2023_4512_MOESM7_ESM.docx]

**Table S1.**SMR analysis of genome-wide cis-eQTLs and uIA/SAH.

| Type of disease | Gene | IV | eQTL size effect | β | OR(95%CI) | Psmr |
| --- | --- | --- | --- | --- | --- | --- |
| uIA | DNMT3A | rs7583409 | -0.1122 | -1.01 | (-2.52-3.25) | 0.014 |
|  | MBD2 | rs2085671 | -0.2272 | 0.52 | (-0.85-4.21） | 0.043 |
| SAH | DNMT3A | rs7583409 | -0.1122 | -0.89 | (-2.19-3.01) | 0.0016 |
