# Supplementary material for: DNA methylation regulator-mediated modification patterns and risk of intracranial aneurysm: a multi-omics and epigenome-wide association study integrating machine learning, Mendelian randomization, eQTL and mQTL data
Source: J Transl Med. 2023 Sep 23;21:660. doi: 10.1186/s12967-023-04512-w (PMC10518114; doi:10.1186/s12967-023-04512-w)
Supplement: Supplementary file 8 — Additional file 8: Table S2. SNP heritability estimates. [file 12967_2023_4512_MOESM8_ESM.docx]

Table S2. SNP heritability estimates.

| Genes | outcome | No.SNP | Method | OR(95%CI) | P |
| --- | --- | --- | --- | --- | --- |
| DNMT1 | SAH | 1 | IVW | (0.090-0.879) | 0.029 |
| DNMT3B | uIA | 1 | IVW | (1.011-8.437) | 0.048 |
